# Relapse in alcohol dependence is characterized by disrupted modular brain network organization

**DOI:** 10.1007/s00406-026-02198-x

**Published:** 2026-01-28

**Authors:** Justin Böhmer, Michael Marxen, Ilya M. Veer, Maria Garbusow, Marco Bottino, Ulrich S. Zimmermann, Michael N. Smolka, Andreas Heinz, Eva Friedel, Christine Heim, Johann D. Kruschwitz, Henrik Walter

**Affiliations:** 1 Department of Psychiatry and Psychotherapy CCM, Charité – Universitätsmedizin Berlin, Corporate Member of Freie Universität Berlin, Humboldt-Universität zu Berlin, Berlin Institute of Health, Berlin, Germany; 2 Institute of Medical Psychology, Charité – Universitätsmedizin Berlin, Corporate Member of Freie Universität Berlin, Humboldt-Universität zu Berlin, Berlin Institute of Health, Berlin, Germany; 3 Department of Psychiatry and Psychotherapy, Technische Universität Dresden, Dresden, Germany; 4 Department of Developmental Psychology, University of Amsterdam, Amsterdam, The Netherlands; 5 Department of Psychology, MSB Medical School Berlin, Berlin, Germany; 6 Department of Addiction Medicine and Psychotherapy, kbo-Isar-Amper-Klinikum München- Ost, Haar, Germany; 7 German Center for Mental Health, Berlin Potsdam Partner Site, Berlin, Germany; 8 NeuroCure Cluster of Excellence, Berlin, Germany

**Keywords:** Alcohol use disorder, Connectomics, Community, Modularity, Relapse, Resting-state fMRI

## Abstract

**Background:**

Patients suffering from alcohol dependence (AD) experience high relapse rates. Prior studies investigating the organization of resting-state functional connectivity networks in AD using graph theory typically focused on alterations of the whole network (macroscale) or on aberrations of single brain regions (microscale). However, little is known about the complex dynamics and interactions among different brain regions and neural systems, i.e. the network organization at mesoscale.

**Methods:**

To investigate mesoscale network alterations, we applied a data-driven community detection algorithm to identify the modular structure of functional brain networks and assess its association with relapse over a 12-month follow-up period in alcohol-dependent patients (relapsers, REL, *n* = 59; abstainers, ABS, *n* = 28) and age- and sex-matched controls (CON, *n* = 83).

**Results:**

Our results reveal differences in the modular organization in REL, marked by a fragmentation and reorganization of major functional modules. Across individuals, functional modules of REL exhibited higher modular variability, particularly in brain regions associated with behavioral and emotional regulatory processes. Conversely, prefrontal reward-related brain regions were more central for inter-module communication in REL, emerging as functional brain hubs. Furthermore, higher overall modular variability significantly predicted time to relapse during follow-up.

**Conclusion:**

Collectively, our results shed light on potential neural substrates of relapse risk in alcohol dependence, which may foster the development of targeted interventions to promote sustained abstinence.

**Supplementary Information:**

The online version contains supplementary material available at 10.1007/s00406-026-02198-x.

## Introduction

Alcohol dependence (AD) represents a major public health concern [1, 2], characterized by a persistent cycle of craving, intoxication, and withdrawal, often leading to repeated relapses [3]. Although significant advances have been made in addiction research and treatment, relapse rates remain high, with up to 75% of patients relapsing within one year following inpatient care [4], highlighting the need to better understand neural mechanisms of relapse vulnerability to inform strategies supporting long-term abstinence.

Resting-state functional connectivity, as measured by functional magnetic resonance imaging (fMRI), offers a valuable means to explore the brain’s intricate network architecture [5]. Specifically, functional brain networks are organized across multiple topological scales, including the whole-brain architecture at macroscale, the formation and interaction of functional subnetworks at mesoscale, and region-specific properties of individual nodes at microscale [6]. Network measures derived at each topological scale offer crucial insights into the brain’s functional architecture in both health and disease, including alcohol dependence [7–9]. Many studies typically focus on alterations of the whole network (macroscale) or on aberrations of single brain regions (microscale). For instance, in the same sample analyzed in this study, we previously demonstrated increased brain-wide functional segregation and reduced functional integration (macroscale) as well as decreased regional centrality in widespread regions of the brain (microscale) in prospectively relapsing alcohol-dependent patients, which predicted time to relapse during a 12-months follow-up period [10]. However, these findings provide only limited insight into the dynamic interplay across distributed brain regions and neural systems that may be related to relapse risk in alcohol dependence. Indeed, previous research indicates that many neurological and psychiatric conditions are characterized by altered dynamics within specific functional subnetworks, rather than by isolated regional disruptions or global changes at the whole-brain level [11, 12]. Therefore, we seek to expand our previous work by investigating brain network alterations at mesoscale, which could provide more specific insights into underlying disease mechanisms, potentially revealing novel biomarkers and therapeutic targets overlooked at other scales.

One popular approach for the study of mesoscopic properties is to investigate the intrinsic community configuration, or modular structure, of the brain [13]. Modules, or communities, are defined as groups of brain regions with dense within-group connectivity and comparatively sparse connections to other regions, thereby facilitating a balance of functional segregation and integration [14]. The extent to which the brain can be partitioned into distinct, non-overlapping modules provides a measure of its overall modularity [15].

Several studies from preclinical alcohol research revealed effects of alcohol exposure, withdrawal and re-exposure on brain network modularity in different animal models [16–18]. In humans with alcohol dependence, however, research on brain network modularity, specifically in relation to relapse risk, remains scarce. One study compared the intrinsic community configuration between patients with alcohol use disorder (AUD) and controls at baseline and at three-months follow-up. Overall modularity was unaltered in those that relapsed relative to either abstainers or controls [19]. However, the observed community structure was relatively coarse, with only three brain modules detected across groups at baseline. This pattern is likely attributable to the resolution limit, a feature inherent to many frequently applied community detection algorithms that restricts the detection of smaller-scale modules [20], putatively obscuring subtler, yet equally meaningful, changes in mesoscale network structure.

In contrast, novel community detection approaches based on the concept of Surprise have been shown to overcome the resolution limit and enable the concurrent identification of modules at different levels of granularity [21], thereby providing a richer description of the network’s complex organization [22]. Moreover, the finer modular decomposition entails improved identification of key regions crucial for communication within the network’s global modular architecture, so-called hubs [23, 24]. An extension of the Surprise community detection for weighted networks, called Asymptotical Surprise, has been validated in synthetic and real-world data [23] and has already been used for the study of brain network modularity in different neuropsychiatric disorders [25, 26].

Consequently, in the present study, we apply an unbiased, resolution limit-free, data-driven community detection algorithm based on the maximization of Asymptotical Surprise to compare the intrinsic community configurations between relapsers (REL), abstainers (ABS) and socially drinking controls (CON) based on a 12-months follow-up period to gain further insight into the connectomic features of alcohol dependence and relapse vulnerability, building upon our previous analysis [10]. Specifically, we investigate different aspects of mesoscale organization, including the robustness of functional networks, the quality, similarity, and spatial consistency of modular partitions as well as hub assignments across groups. Since Asymptotical Surprise allows more fine-grained investigations of mesoscale network characteristics, we expect significantly lower network robustness, lower modular partition quality, decreased similarity, increased modular variability as well as aberrant hub organizations in alcohol dependence relative to controls. Based on previous work [10, 19], we expect to observe such aberrations particularly in prospective relapsers. Finally, we hypothesize that altered mesoscale network metrics are associated with drinking outcomes and behavioral relapse measures during follow-up, suggesting a potential neural underpinning for relapse risk in alcohol dependence.

## Methods

### Participants

Subjects were recruited within a large bicentric study conducted in Berlin and Dresden investigating predictors for the development and maintenance of alcoholism (NCT01679145). In the present study, 93 patients aged 18 to 65 suffering from alcohol dependence for at least three years according to ICD-10 and DSM-IV-TR criteria (*M* = 11.62 years according to DSM-IV-TR, *SD* = 10.05) were assessed shortly after their detoxification treatment (*M* = 23.23 days, *SD* = 12.63). Additionally, 83 controls with social drinking behavior matched on sex and age were included in the study as a control group. Exclusion criteria included other substance use disorders (except alcohol, nicotine), major psychiatric or neurological disorders, or psychoactive medication. For relapse assessment, AD patients were continuously followed-up for 12 months (48 weeks) after MRI measurement using the Timeline-Follow-Back (TLFB) [27]. Follow-up assessments were completed by 87 AD patients. Relapse was defined as one drinking occasion during the follow-up interval with at least 60 g/48 g of alcohol intake for male and female subjects, respectively, or when the phosphatidylethanol (PEth) levels determined from dried blood spots of venous blood at either 12- or 24-weeks follow-up exceeded 112 ng/ml [28]. According to this definition, our sample consisted of 59 relapsers (REL) and 28 abstainers (ABS). A detailed description of the sample and follow-up procedure can be found in Böhmer et al. [10] and in Table 1. The study was carried out in line with the Declaration of Helsinki and was approved by the local medical ethics committees. All subjects provided informed written consent prior to study participation.

**Table 1:** Demographics and clinical characteristics

Variable (mean ± SD)	AD patients		CON (*n* = 83)	*p*-value (post-hoc comparison)
	REL (*n* = 59)	ABS (*n* = 28)		
Sex m/f	52/7	23/5	70/13	.718^a^
Age in years	45.50 ± 9.56	43.48 ± 11.85	43.40 ± 11.12	.490^b^
Education in years	14.09 ± 3.27	14.63 ± 3.81	15.56 ± 3.19	.010^ce^ (REL < CON)
Mean framewise displacement (FD)	0.17 ± 0.14	0.13 ± 0.07	0.12 ± 0.06	.253^c^
CIDI: Age of first drink	14.66 ± 2.55	15.18 ± 2.55	14.71 ± 2.33	.766^c^
CIDI: Age of first time drunk	16.64 ± 3.07	16.29 ± 3.49	16.71 ± 3.51	.578^c^
CIDI: Age of first binge-drinking episode	30.67 ± 10.16	32.39 ± 12.18	21.54 ± 7.18	< .001^ce^ (REL > CON, ABS > CON)
CIDI: Alcohol consumption per day (g)	178.85 ± 124.71	151.98 ± 91.56	11.74 ± 13.50	< .001^ce^ (REL > CON, ABS > CON)
CIDI: Alcohol consumption per occasion (g)	205.63 ± 108.23	176.79 ± 93.06	44.46 ± 32.94	< .001^ce^ (REL > CON, ABS > CON)
CIDI: Alcohol consumption per binge-drinking episode (g)	289.68 ± 165.99	257.79 ± 123.11	94.45 ± 96.12	< .001^ce^ (REL > CON, ABS > CON)
ADS: Addiction severity	14.85 ± 6.42	14.11 ± 7.29	1.94 ± 2.89	< .001^ce^ (REL > CON, ABS > CON)
OCDS: Craving	12.33 ± 8.46	11.38 ± 8.31	2.87 ± 2.98	< .001^ce^ (REL > CON, ABS > CON)
Time of alcohol abstinence before MRI scan in days	24.58 ± 14.35	20.39 ± 7.31	-	.328^d^
TLFB: Time until relapse in days	130.90 ± 100.32	-	-	-

^a^Chi-square (χ2) test, ^b^One-way ANOVA, ^c^Kruskal-Wallis test, ^d^Mann-Whitney U test, ^e^Bonferroni-corrected for three group comparisons. Abbreviations: REL = relapsing AD patients, ABS = abstaining AD patients, CON = controls, CIDI = Composite International Diagnostic Interview, ADS = Alcohol Dependence Scale, OCDS = Obsessive Compulsive Drinking Scale, TLFB = Timeline-Follow-Back

### Clinical and psychometric assessment

We employed the computerized version of the Composite International Diagnostic Interview (CIDI) [29] for diagnosis and assessment of drinking patterns. Moreover, we administered the Alcohol Dependence Scale (ADS) [30] and the Obsessive Compulsive Drinking Scale (OCDS) [31] to assess alcohol dependence severity and alcohol craving, respectively.

### fMRI acquisition and preprocessing

MRI was performed at 3T Siemens Magnetom Tim Trio MRI scanners with a 12-channel phased-array head coil (Siemens Healthineers, Erlangen, Germany). For each participant, 148 functional images were acquired during resting-state using a gradient-echo echo-planar imaging (GE-EPI) sequence (TR = 2410ms, TE = 25ms, 80° flip angle, 42 axial (ax > cor) slices with 1 mm slice gap, 2 mm slice thickness, FOV = 192 × 192mm^2^, 3 × 3mm^2^ in-plane resolution, bandwidth = 2112 Hz/Px). During acquisition, participants were instructed to relax and to keep their eyes closed without falling asleep. Additionally, high-resolution T1-weighted 3D structural images (MPRAGE, TR = 1900ms, TE = 2.52ms, TI = 900ms, 9° flip angle, isotropic voxel size of 1 × 1 × 1 mm³, bandwidth = 170 Hz/Px) and a B0-field map (TR = 488ms, TE1 = 5.32ms, TE2 = 7.78ms, 60° flip angle, 3 × 3 × 2 mm³ voxel size, bandwidth = 260 Hz/Px) were acquired.

Data preprocessing was performed using the FMRIB Software Library (FSL) v5.0 and included motion and slice-timing correction, brain extraction, spatial smoothing (6 mm FWHM), ICA-based denoising (ICA-AROMA), high-pass filtering (0.008 Hz), co-registration to T1, non-linear normalization to 2 mm MNI space (ANTs), and resampling to 3 mm voxels. Participants with excessive head movements after head motion correction (i.e., mean framewise displacement (FD) > 0.5 mm) were excluded (*n* = 7; CON: *n* = 1, ABS: *n* = 3, REL: *n* = 3).

### Network construction

**Fig. 1:**
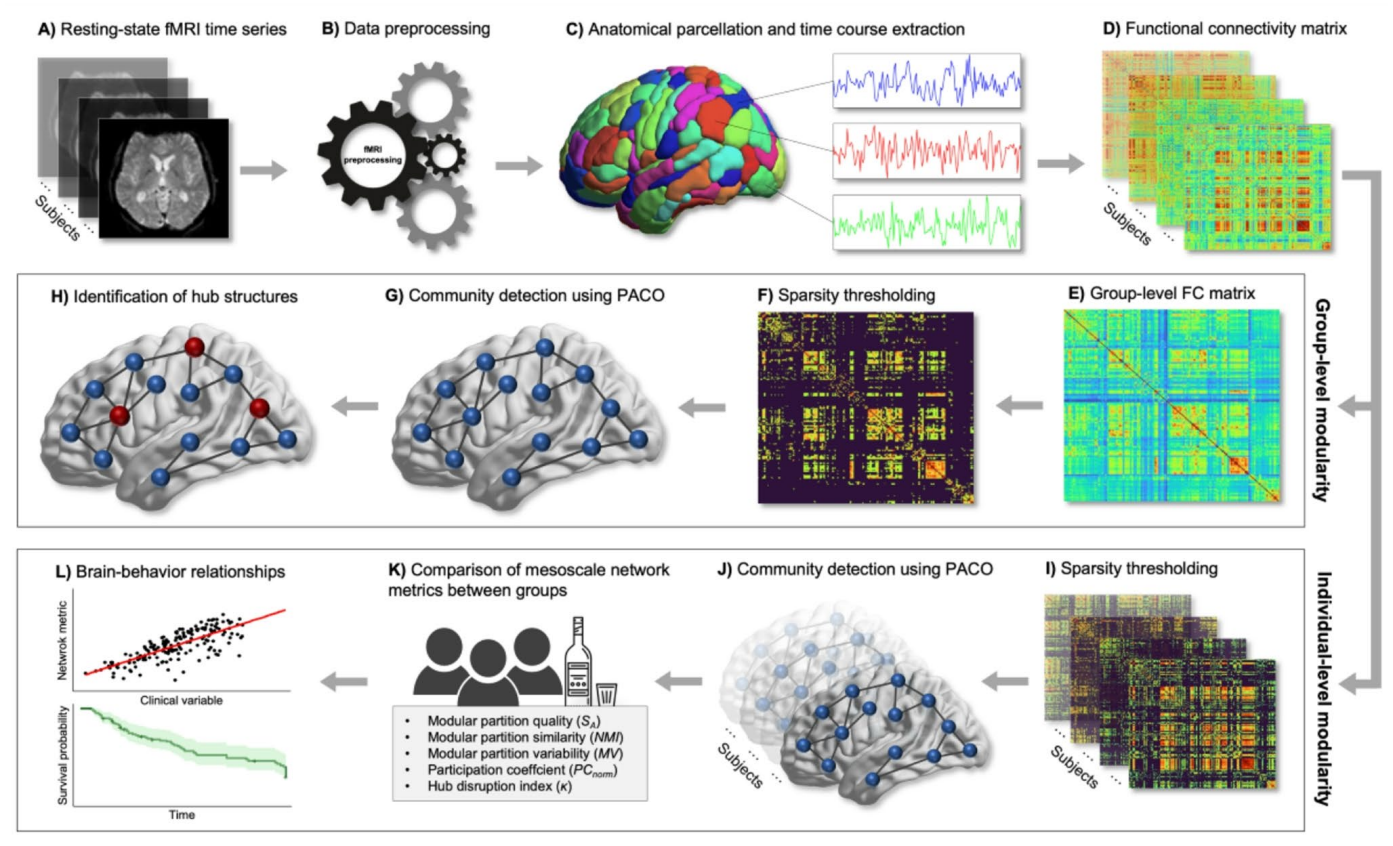
Analysis pipeline. **A** Resting-state fMRI time series were acquired for each participant and **B** subjected to preprocessing (see Methods). **C** Mean time series were extracted for each of the 246 regions of interest (ROI) of the Brainnetome Atlas. **D** Functional connectivity was estimated for each subject by computing the pairwise inter-regional Pearson correlation coefficient between all possible pairs of ROIs. **E** To evaluate modularity on the group-level, first, individual FC matrices were Fisher z-transformed and averaged by group. **F** Network sparsification based on the percolation point was performed to remove spurious edges. **G** Community detection based on the maximization of asymptotical surprise, performed via the PACO algorithm, was applied to the thresholded group-level FC matrices. **H** Hub nodes were identified based on the normalized participation coefficient. **I** To evaluate modularity on the individual-level, we applied the thresholding procedure to each individual’s FC matrix in order to **J** compute the modular configuration for each subject. **K** Individual-level network properties were calculated to compare different aspects of mesoscale network organization between groups. **L** Finally, brain-behavior relationships between altered network metrics and drinking variables were investigated using both correlational and survival analyses

Network nodes were obtained based on the Brainnetome Atlas [32], resulting in a network of 246 nodes. We selected the Brainnetome Atlas as it provides a connectivity-based parcellation framework that offers comprehensive cortical and subcortical coverage [32]. For each node, we extracted the mean time series from all corresponding voxels and estimated functional connectivity by computing the pairwise inter-regional Pearson correlation coefficients between all possible pairs of nodes. To compare the mesoscale network architecture between groups, we investigated the modular organization at both the group-level and the individual-level [33]. See Fig. 1 for an overview of the analysis pipeline. To derive group-level connectivity matrices, individual matrices were Fisher z-transformed and averaged by group. Network sparsification, i.e. removal of edges with low z-values, was conducted up to the percolation point, corresponding to the largest threshold that maintains a path between any two of the 246 network nodes. Following this thresholding procedure, only positive edges remain. The percolation point reflects the optimal balance between genuine connections and the removal of spurious edges introduced by noise, thereby maximizing the ability to retrieve the network’s modular structure [34, 35]. This results in slightly different sparsity thresholds for each group matrix and for each individual subject matrix (see Fig. S1). To account for potential influences of varying sparsity thresholds and given the observed correlations between percolation-based network density and several mesoscale metrics (Fig. S2), we included the edge density after percolation thresholding as an additional covariate in our control analyses and conducted robustness checks to verify the replicability of our findings using proportional thresholding. Finally, the percolation-thresholded connectivity matrices for each group and each individual were used as input for the community detection algorithm.

### Community detection based on asymptotical surprise

The modular organization was determined by maximization of the Asymptotical Surprise (*S*_*A*_) fitness function [21, 23]. *S*_*A*_ is an information-theoretic metric quantifying the degree to which the number of intra-modular edges from a given modular partition deviates from the number of intra-modular edges expected by chance in a random graph [23, 36]. A large *S*_*A*_-value suggests that the given modular partition is highly informative and unlikely a product of random variation. To perform module detection based on the maximization of *S*_*A*_, we used the Partitioning Cost Optimization (PACO) algorithm (https://github.com/CarloNicolini/paco), with an agglomerative optimization method and Asymptotical Surprise as the quality function to maximize. PACO enables an unbiased identification of brain network modules in a fully data-driven way, as it does not depend on prior assumptions about the number of communities. Moreover, PACO has been shown to be quasi-resolution limit-free [23], allowing for the identification of modules at different scales and levels of granularity. As PACO is a non-deterministic algorithm that yields slightly different modular partitions in each run, we performed 5,000 runs of PACO and selected the partition with the highest *S*_*A*_-value. Asymptotical Surprise does not suffer from degeneracy of nearly optimal solutions, whereby similarly large *S*_*A*_-values correspond to substantially different modular partitions [23]. Consequently, it is not necessary to apply a consensus approach over various partitions with comparably high values of the fitness function [37].

### Mesoscale network metrics

We investigated different aspects of modularity to assess potential differences in the mesoscale network organization between groups. Importantly, we analyzed both group-level and individual-level network alterations.

1. *Percolation-based network sparsification.* First, we investigated the robustness of functional networks by comparing the percolation point between groups. Percolation analyses were conducted on both the group-level and individual-level functional connectivity matrices.
2. *Modular decomposition.* Second, we investigated whether modular partitions differ between groups by calculating the normalized mutual information (NMI) index [38]. NMI provides a measure of agreement between two network partitions, with higher values indicating greater similarity. In the present study, we used an extension of NMI that accounts for differences in the number of modules between groups [39]. We calculated NMI to quantitatively describe the disparity of group-level modular partitions. Moreover, we conducted a permutation procedure to test for significant differences between groups based on individual-level community partitions (see Extended Methods in Supplementary Information). Modular partitions were considered significantly different between groups if *p* < .05.
3. *Partition quality and modular fragmentation.* Third, we evaluated the quality of modular partitions based on the individual-level community configurations by comparing the subject-specific Asymptotical Surprise values (*S*_*A*_) between groups. Likewise, we compared the number of modules, the average module size and the average spatial extent of modules (i.e., module radius [40]) between groups to assess the presence of modular fragmentation.
4. *Modular variability.* Fourth, we explored differences in the spatial variability of network modules across subjects by computing modular variability (*MV*) [33] (see Extended Methods in Supplementary Information). We compared the mean *MV* value for each region of the brain and the mean across all brain regions between groups.
5. *Hub assignments and hub disruption index.* Lastly, we explored differences in hub assignments across groups. In this regard, we first identified hub regions based on the normalized participation coefficient (*PC*_*norm*_) and then calculated the hub disruption index *κ* [41] (see Extended Methods in Supplementary Information). For inference statistical testing, *κ* was estimated for each subject by contrasting the individual-level *PC*_*norm*_ with the group-averaged *PC*_*norm*_ of CON and comparing it between groups.

### Statistical analysis

All group comparisons of individual-level mesoscale network metrics were carried out using non-parametric permutation-based ANOVA while controlling for age, sex, site, years of education and mean FD. For each metric, we first computed the empirical one-way ANOVA F-statistic for differences between groups. We then randomly permuted group labels 10,000 times, recalculated the F-value for each permutation, and derived the p-value as the proportion of permuted F-values equal to or greater than the empirical value. Metrics showing significant group differences (*p* < .05) were followed up with permutation-based post-hoc tests. Here, we computed the empirical group-wise differences using two-sample t-tests and repeated the label-shuffling procedure (10,000 permutations) to obtain permutation-based p-values. Multiple testing correction was applied using Bonferroni correction. For global metrics (i.e., one value per subject), we accounted for the number of group contrasts (*α* = 0.05 / 3 = 0.017). For regional metrics (i.e., one value per region per subject), we corrected based on the number of brain regions (*α* = 0.05 / 246 = 0.0002). To account for differences in sparsity thresholds, we included edge density after percolation as a covariate in our control analyses.

### Brain-behavior relationships

We performed partial correlation analyses between significantly altered brain variables and clinical and drinking outcomes, accounting for age, sex, site, years of education and mean FD. In case of non-normality of either clinical or brain variables as determined by Shapiro-Wilk tests (*p* < .05), we computed Spearman’s partial rank correlation. To account for multiple testing, Bonferroni correction was applied, controlling for the number of network metrics included in the correlation analyses.

Moreover, we computed Cox proportional hazards models to determine the relation between altered mesoscale network metrics and time to relapse during follow-up, defined as the number of days from baseline to the first relapse event. To this end, we employed separate univariate models for each altered network metric. As a sensitivity analysis, multivariate models were fitted for those metrics that were significant in the univariate analyses (corrected for the number of univariate models tested), adjusting for age, sex, site, education, and mean FD. The proportional hazards assumption was evaluated using scaled Schoenfeld residuals, and all network measures and covariates were standardized (z-scores) prior to analysis to improve interpretability and comparability of hazard ratios (HR).

## Results

### Mesoscale network metrics

#### Percolation-based network sparsification

Network sparsification based on the percolation threshold was performed for each group and each individual separately. At the group-level, a total of 5,392 edges were retained in CON, corresponding to 17.89% network density. In the patient groups, a total of 6,383 edges (21.18% network density) were kept in ABS and 2,262 edges (7.51% network density) in REL after percolation thresholding (Fig. S1). At the individual level, we observed significantly lower network density in REL compared to CON after thresholding (*p* = .044, Table 2, Fig. S3), mirroring differences in network density from group-averaged matrices.

**Table 2:** Comparison of mesoscale network metrics between groups

Network variable (mean ± SD)	AD patients		CON (*n* = 83)	*p*-value^a^ (post-hoc comparison^b^)
	REL (*n* = 59)	ABS (*n* = 28)		
Percolation-based network density	0.36 ± 0.18	0.40 ± 0.17	0.43 ± 0.19	0.036 (REL < CON)
Modular partition quality (*S*_*A*_)	2705.03 ± 1054.72	2474.49 ± 864.94	2372.57 ± 954.41	0.263
Number of modules	21.98 ± 6.40	20.93 ± 5.08	20.88 ± 6.47	0.718
Module size	12.16 ± 3.72	12.65 ± 4.05	12.96 ± 4.24	0.593
Module radius	0.81 ± 0.07	0.82 ± 0.05	0.83 ± 0.06	0.285
Modular variability (*MV*)	0.95 ± 0.01	0.94 ± 0.02	0.94 ± 0.02	< 0.001 (REL > CON, REL > ABS)
Participation coefficient (*PC*_*norm*_)	0.67 ± 0.05	0.69 ± 0.05	0.69 ± 0.04	0.002 (REL < CON)
Hub disruption index (*κ*)	-0.22 ± 0.54	-0.07 ± 0.43	0.00 ± 0.50	0.031 (REL < CON)

Note: Metrics are derived from individual-level functional connectivity matrices. ^a^Permutation-based ANOVA (10,000 permutations), controlling for age, sex, site, education years and mean framewise displacement (FD). ^b^Post-hoc permutation-based t-tests with Bonferroni-adjusted *p*-value, corrected for the number of group comparisons (*α* = 0.05 / 3 = 0.017). Abbreviations: CON = controls, ABS = abstaining AD patients, REL = relapsing AD patients

#### Modular decomposition

**Fig. 2:**
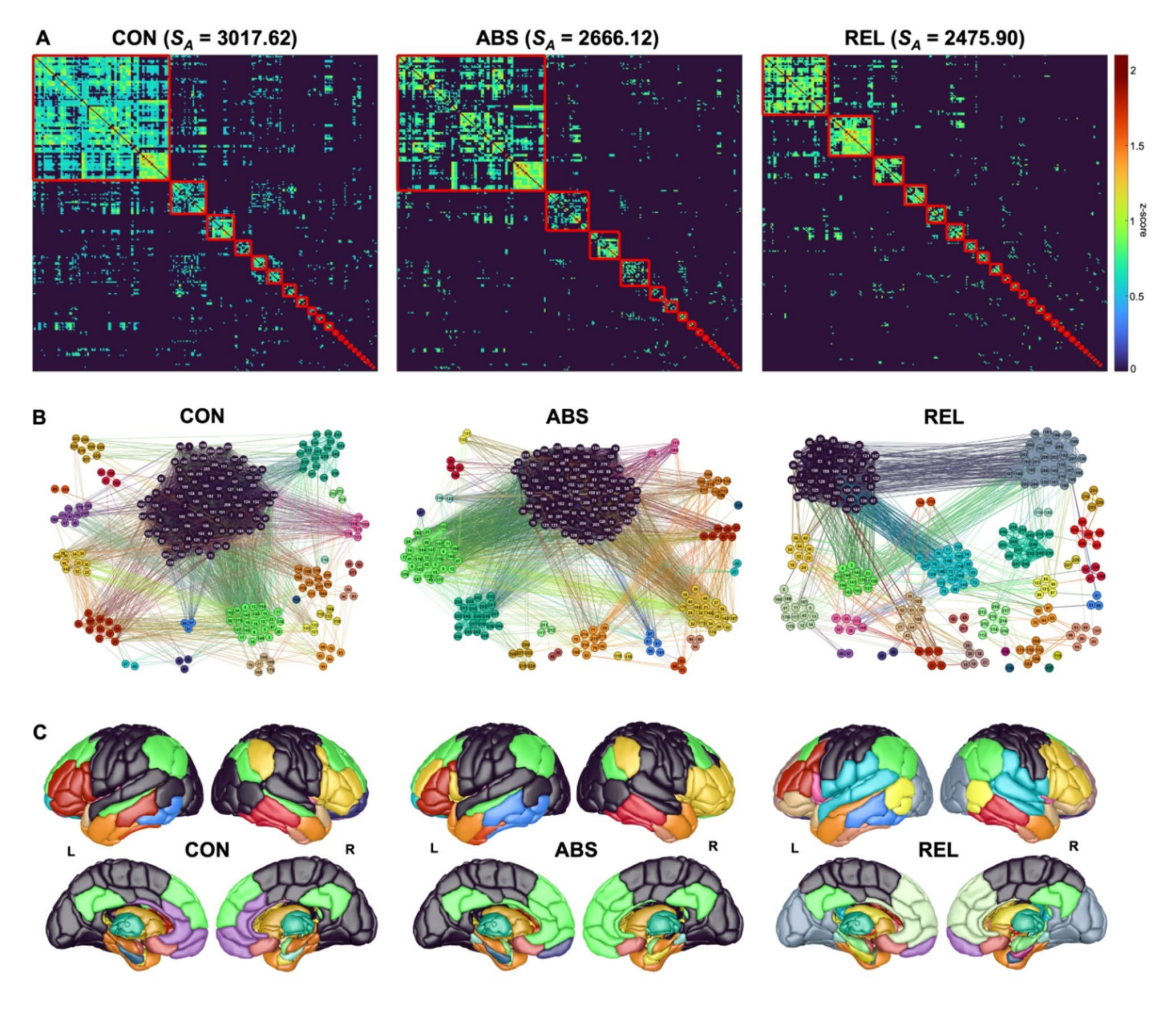
Group-level modular organization. **A** Percolation-thresholded region-by-region functional connectivity matrices sorted by module membership obtained by maximizing Asymptotical Surprise (*S*_*A*_) for controls (CON), relapsing AD patients (REL) and abstaining AD patients (ABS). Modules are demarcated by a red line. The number of modules is 23, 21 and 31 for CON, ABS and REL, respectively. The *S*_*A*_ value indicates the modular partition quality. **B** Visualization of intra- and inter-modular connectivity. Nodes are organized into color-coded modules according to the group-level partition. Node numbers refer to Brainnetome Atlas regions. **C)** Surface rendering of brain modules. Brain visualizations were created using the Scalable Brain Atlas Composer (https://scalablebrainatlas.incf.org/composer/)

Based on the group-level percolation-thresholded connectivity matrices, the optimal modular partition as identified by PACO comprised 23 functional modules in the CON group (*S*_*A*_ = 3017.62), 21 modules in the ABS group (*S*_*A*_ = 2666.12) and 31 modules in the REL group (*S*_*A*_ = 2475.90), see Fig. 2. Qualitative examination of group-level modular structure revealed relatively more similar organization in ABS vs. CON (*NMI* = 0.73) than in REL vs. CON (*NMI* = 0.58) and ABS vs. REL (*NMI* = 0.51). To test for significance, we implemented a permutation procedure based on the individual-level modular structure, revealing significantly different modular configurations in REL compared to CON (*p* = .042). We found no significant differences in community assignments between REL vs. ABS (*p* = .214) and ABS vs. CON (*p* = .139).

**Fig. 3:**
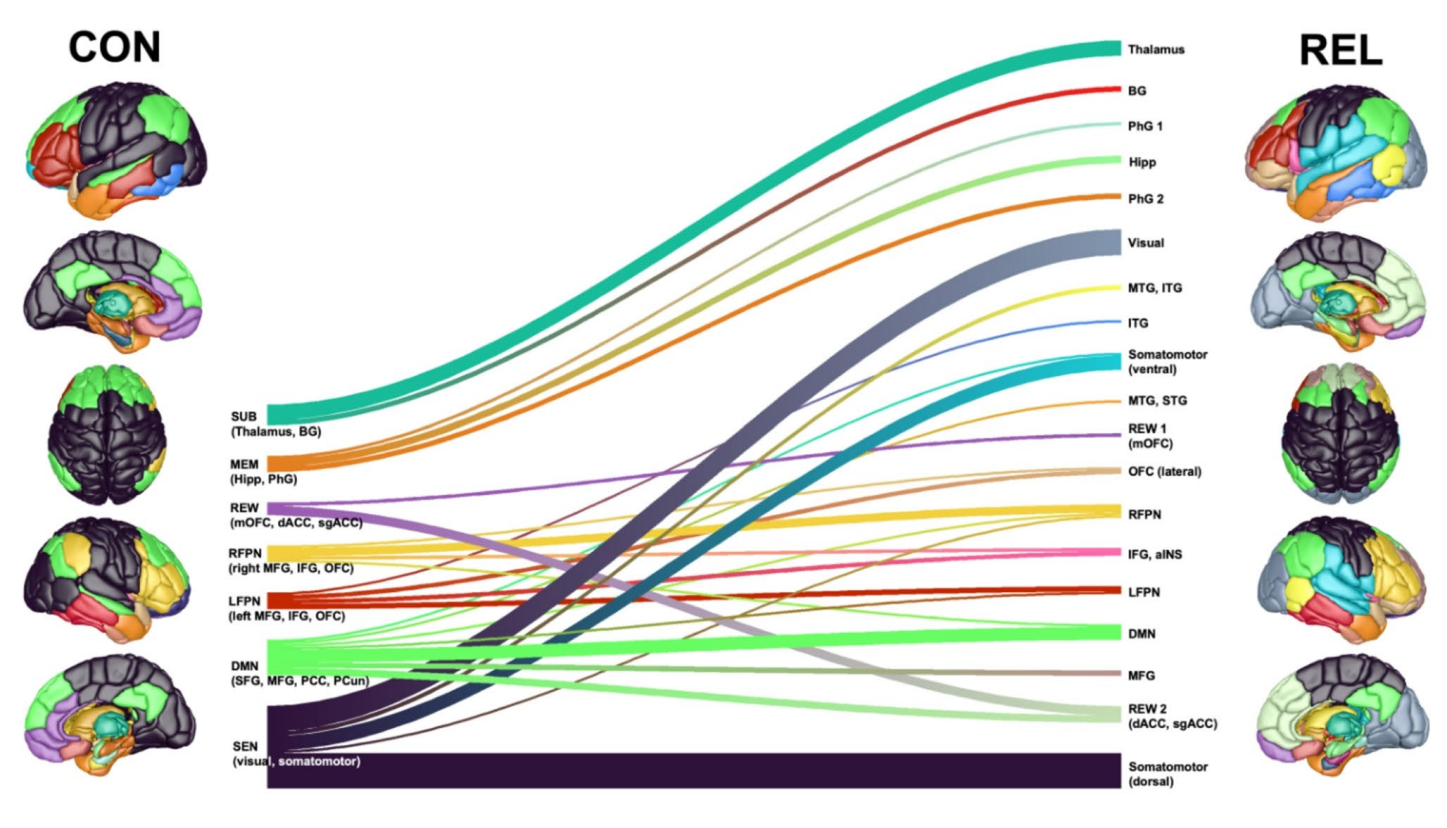
Fragmentation and reorganization of functional modules in relapsing AD patients (REL) compared to controls (CON). The seven largest functional modules from the group-level modular partition in the CON group fragment into 19 smaller submodules in the REL group. Abbreviations: aINS = anterior insula, BG = basal ganglia, dACC = dorsal anterior cingulate gyrus, DMN = default mode network, Hipp = hippocampus, IFG = inferior frontal gyrus, ITG = inferior temporal gyrus, LFPN = left frontoparietal network, MEM = memory network, MFG = middle frontal gyrus, MTG = middle temporal gyrus, OFC = orbitofrontal cortex, PCC = posterior cingulate cortex, PCun = precuneus, PhG = parahippocampal gyrus, REW = reward network, RFPN = right frontoparietal network, SEN = sensory network, SFG = superior frontal gyrus, sgACC = subgenual cingulate gyrus, STG = superior temporal gyrus, SUB = subcortical network

To further characterize the difference in modular configurations between REL and CON, we computed the spatial correlation [42] between the group-level community partition in the CON group and five large-scale canonical brain network templates and assigned a label for each module in CON based on the largest spatial correlation and the core brain regions constituting the module (see Tab. S1). Afterwards, we evaluated the correspondence of these functionally-defined modules between CON and REL, focusing on the seven largest modules in CON (i.e., modules consisting of at least 10 brain regions) (Fig. 3). Most strikingly, sensory regions that formed a unified module in CON were split into three distinct components in REL, corresponding to visual, dorsal somatomotor, and ventral somatomotor modules. Similarly, the left and right fronto-parietal networks fragment into four smaller modules, respectively. Moreover, the medial orbitofrontal cortex in REL is split from the dorsal and subgenual anterior cingulate cortex, which constitutes the reward-related module in CON. The memory-related module, encompassing mainly the hippocampus and parahippocampal gyrus, fragments into one hippocampal module, one anterior, and one posterior parahippocampal module. Lastly, the subcortical module divides into a thalamic module and a basal ganglia module (Fig. 3).

#### Partition quality and modular fragmentation

We observed no significant differences between groups regarding the quality of modular partitions, the number of modules, the average module size and the average module radius (Table 2, Fig. S3). Additionally accounting for the percolation-based network density or using a proportional sparsity thresholding procedure did not alter the direction or significance of the effects.

#### Modular variability

**Fig. 4:**
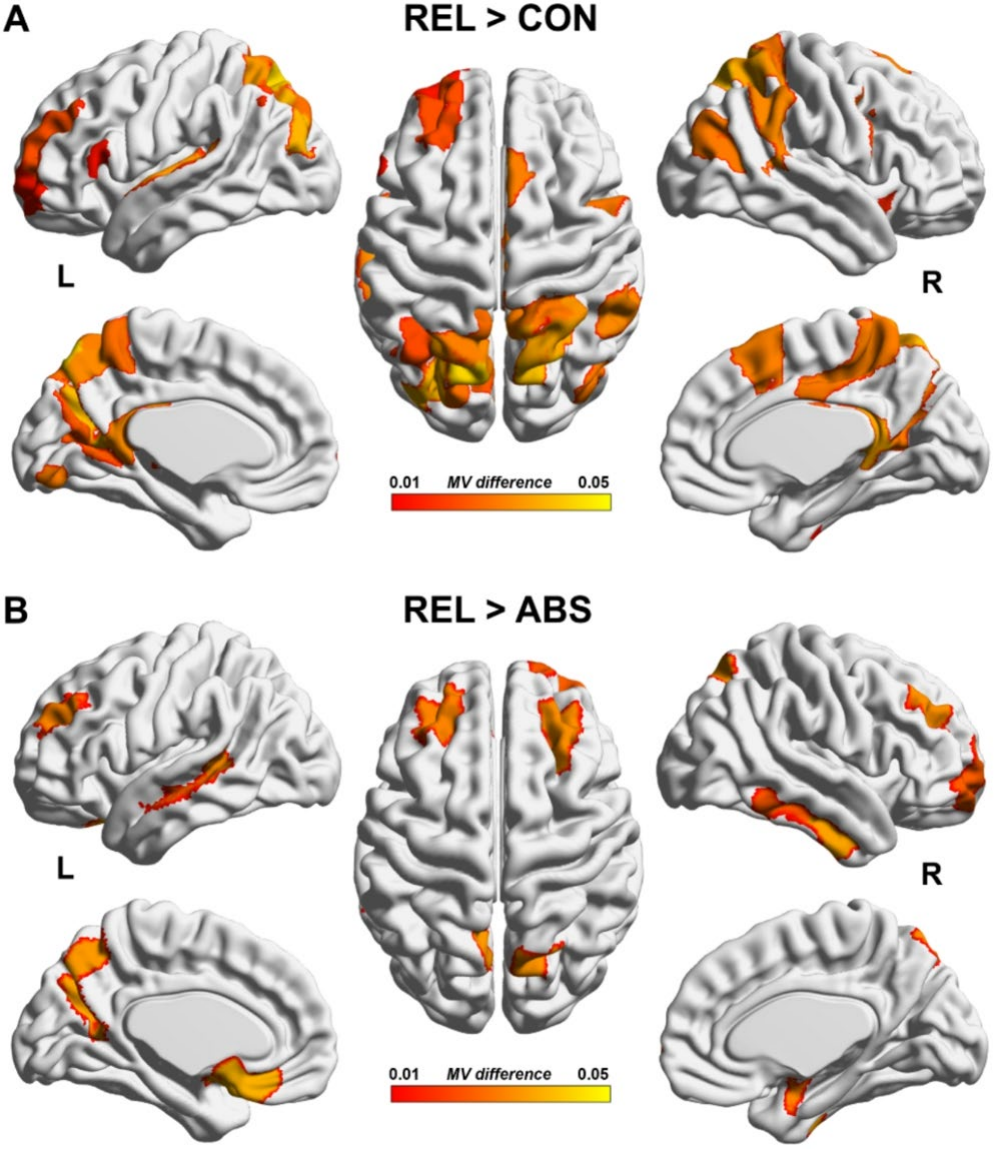
Differences in inter-subject modular variability (MV). **A** Relapsing AD patients (REL) exhibit increased spatial variability of modular assignments as compared to controls (CON), implicating widespread regions across the brain (see Tab. S2). **B** Similarly, REL display increased variability of modular assignments as compared to abstaining AD patients (ABS), specifically in bilateral dorsolateral prefrontal cortex, left medial orbitofrontal cortex, right ventromedial prefrontal cortex, right inferior and left middle temporal gyrus, right superior parietal lobule, left precuneus and right lateral amygdala (see Tab. S3). Significant regions are based on non-parametric permutation tests (10,000 permutations, Bonferroni-corrected)

Across all brain regions, modular partitions in REL were less spatially consistent, reflected by overall higher modular variability (MV) in REL compared to CON (*p* < 0.001) and in REL relative to ABS (*p* = 0.021) (Table 2, Fig. S3). This finding replicated when additionally controlling for the percolation-based sparsity threshold (REL vs. CON: *p* < 0.001; REL vs. ABS: *p* = 0.046) and when using a proportional sparsity thresholding procedure (Fig. S4). At the level of individual brain regions, REL exhibited greater MV compared to CON in the left dorsolateral prefrontal cortex, superior and inferior parietal cortex, posterior cingulate cortex, and precuneus (see Fig. 4A and Tab. S2). In addition, REL exhibited greater MV compared to ABS in the bilateral dorsolateral prefrontal cortex, left medial orbitofrontal cortex, right ventromedial prefrontal cortex, left middle and right inferior temporal gyrus, right superior parietal lobule, left precuneus and right lateral amygdala (see Fig. 4B and Tab. S3).

#### Hub assignments and hub disruption index

**Fig. 5:**
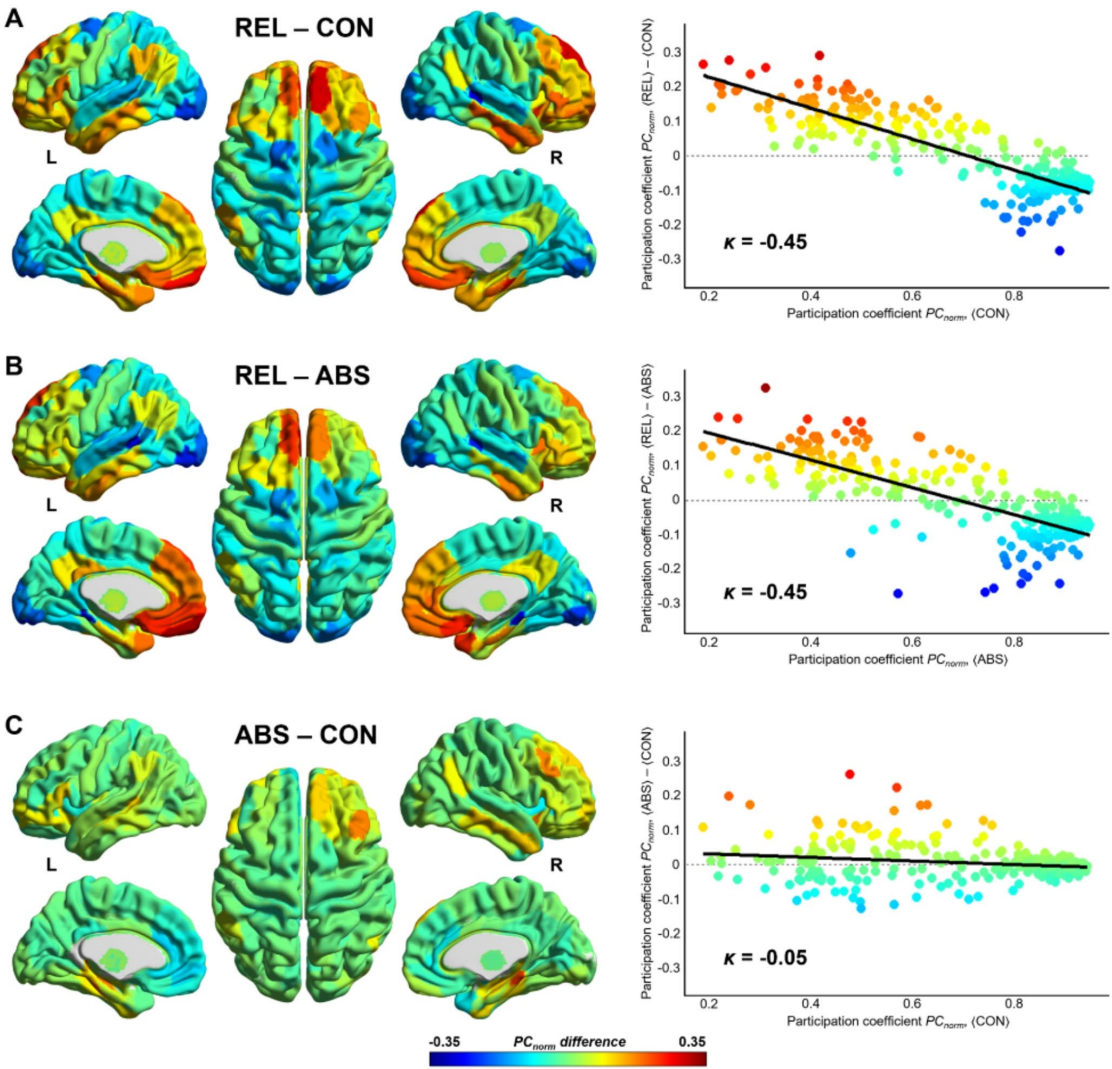
Hub disruption in alcohol dependence (AD). Based on the group-level FC matrices, we computed the normalized participation coefficient (*PC*_*norm*_) and calculated the difference in *PC*_*norm*_ for each brain region between **A** prospective AD relapsers and controls (REL – CON), **B** prospective AD relapsers and prospective AD abstainers (REL – ABS), and **C** prospective relapsers and controls (ABS – CON). Left: Surface representation of the difference in *PC*_*norm*_ between groups. Right: The *PC*_*norm*_ of each node from a reference group was plotted against the difference in *PC*_*norm*_ between the reference group and the group under study. The slope of the black line fitted to the scatter plot indicates the hub disruption index *κ*, a measure of hub reorganization between groups. Contrasting REL – CON and REL – ABS reveals a steep negative slope (*κ* = -0.45 in both cases), while ABS – CON yields a flat regression line (*κ* = -0.05), indicating a reorganization of functional brain hubs in REL. Colors in the left panel correspond to the colors in the right panel

We computed the hub disruption index *κ* for each group contrast based on the *PC*_*norm*_ for each brain region derived from group-level FC matrices (Fig. 5). Our results show a substantial reorganization of functional hubs in REL compared to both CON and ABS (*κ* = -0.45, respectively). Particularly, REL exhibited increased hubness in dorsomedial prefrontal, orbitofrontal, anterior and posterior cingulate, parahippocampal and inferior temporal cortex. Conversely, decreased hubness was observed in supplementary motor area, occipital polar cortex and superior temporal regions (Fig. 5A, B). ABS and CON exhibited largely consistent hub architectures (Fig. 5C).

To test for statistical significance, between-group comparisons of *κ*, estimated from individual-level *PC*_*norm*_, were carried out. These results corroborated our findings from the group-averaged data, revealing a significant disruption of functional brain hubs in REL compared to CON (*κ* = -0.22, *p* = .034, Table 2, Fig. S3). The difference in *κ* between REL and ABS, however, was not significant (*κ* = -0.20, *p* = .149).

### Brain-behavior relationships

Increased variability of modular assignment of the left posterior parietal thalamus (Tha_L_8_5) was associated with increased craving in REL, indicated by a positive correlation with the OCDS total score (*r* = .44, *p* = .049, Bonferroni-corrected). No significant associations were observed between other mesoscale network metrics and clinical variables after Bonferroni correction (Tab. S4).

**Fig. 6:**
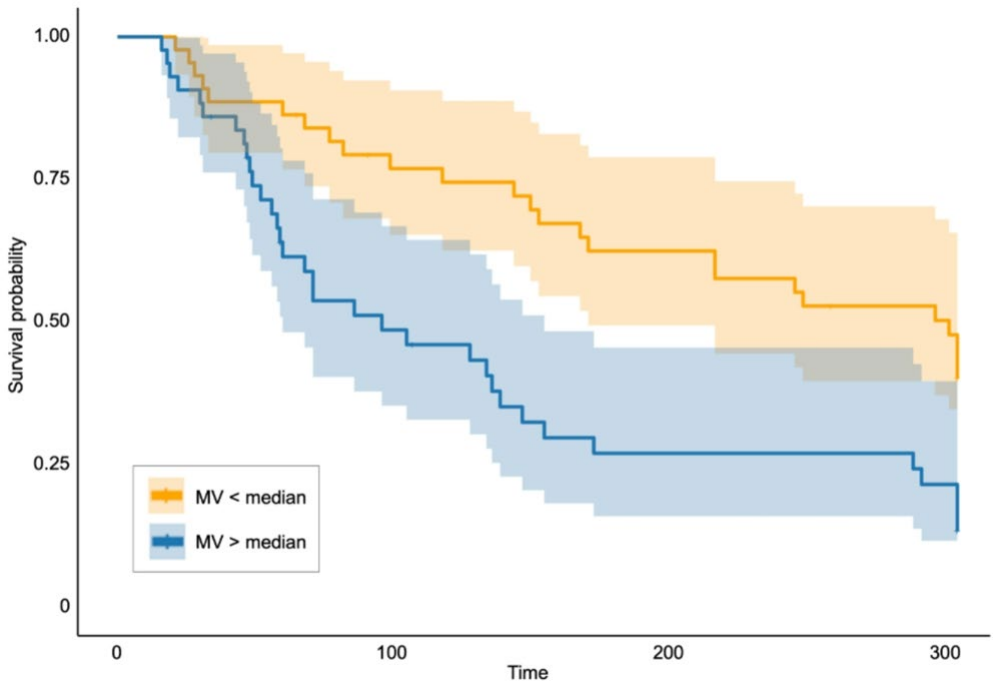
Kaplan–Meier survival curves illustrating time to relapse among patients with alcohol dependence, stratified into high (blue) and low (orange) modular variability groups based on a median split

Univariate survival analyses indicate that, among all altered mesoscale network metrics, only global MV significantly predicted time to relapse in AD patients (Overall model: *χ*^*2*^(1) = 10.91, *p*_*Model*_ < 0.001, *Harrell’s C* = 0.63; HR = 1.55, 95%CI = 1.19–2.02, *p*_*modular_variability*_ = 0.001), see Tab. S5. The corresponding Kaplan-Meier survival curves for high vs. low MV groups are shown in Fig. 6. Adjusting for the effects of age, sex, site, years of education and mean FD in a subsequent multivariate model, modular partition variability still predicted time to relapse (Overall model: *χ*^*2*^(5) = 11.85, *p*_*Model*_ = 0.037, *Harrell’s C* = 0.63; HR = 1.56, 95%CI = 1.18–2.05, *p*_*modular_variability*_ = 0.002), while no other variable significantly contributed to the prediction, see Tab. S6.

### Robustness and sensitivity checks

To ensure that findings were not dependent on analytic choices or sample characteristics, we conducted a series of robustness checks, including split-half reliability of mesoscale network metrics, site-stratified analyses, consensus-based community detection strategies, inclusion of clinical covariates, and replication with a different brain parcellation. Results from these analyses support the reliability of the analytical approach and robustness of the main findings (see Extended Results in Supplementary Information).

## Discussion

While previous studies on the topological organization of brain networks in AD primarily focused on micro- and macroscopic characteristics, the present study investigates alterations at mesoscale by assessing the intrinsic community configuration in prospective relapsers (REL), future abstainers (ABS) and controls (CON) based on a 12-months follow-up interval. Our results demonstrated an overall aberrant modular organization in REL but not in ABS, characterized by a fragmentation and reorganization of major functional modules compared to CON. Furthermore, we observed a significant reconfiguration of functional brain hub distributions as well as larger modular variability in REL. Importantly, higher heterogeneity of modular assignments significantly predicted time to relapse, suggesting a potential neural basis for relapse risk in AD.

### Lower percolation threshold may suggest decreased network robustness in relapsers

To obtain the intrinsic community configurations, functional connectivity matrices were sparsified based on the percolation point. Interestingly, prospective relapsers exhibited lower network density upon reaching the percolation point, meaning that fewer functional connections are necessary to maintain the network in a fully connected state. In this regard, percolation analysis has been proposed as an indicator for network robustness, or redundancy of the network. Network redundancy rests upon the notion that two nodes can be linked by multiple apparently redundant paths to provide alternative propagation ways in the event of perturbations, thereby ascertaining network functionality [43, 44]. Following this rationale, networks in controls and abstainers would exhibit greater resilience in the event of random attacks, whereas in prospective relapsers nodes would disconnect more rapidly from the rest of the network to form isolated subnetworks. Consequently, our results suggest lower network redundancy, and therefore elevated vulnerability to network disruptions, in prospective relapsers. In this regard, similar findings of lower network redundancy have been reported in schizophrenia [45] and discussed as neural underpinning for other psychiatric brain disorders [46].

### Fragmentation of major functional brain modules in relapsers

In correspondence with previous studies, we investigated the modular organization at the group-level to identify general organizational principles at the level of the population [33, 47]. Consistent with these studies, we identified several distinct yet functionally interconnected modules in all three groups, including sensory, default mode, fronto-parietal, reward, memory, and subcortical systems. However, despite generally similar modules, the modular partitions in relapsers were significantly different from the partitions observed in healthy controls. Particularly, relapsers exhibited a greater number of modules, which may suggest a breakdown of larger cohesive structures into smaller, more fragmented submodules. This pattern aligns with our finding of greater segregation into specialized subnetworks in relapsers [10]. The most notable difference was found within the sensory system: whereas in controls and abstainers, this module included both visual and somatomotor areas, in relapsers it split into separate visual, dorsal somatomotor, and ventral somatomotor modules. Decoupling between individual nodes from visual and sensorimotor systems has been reported in relapsing alcohol-dependent patients already in our previous work [10]. Moreover, weaker functional connectivity between lateral occipital cortex and somatomotor regions has been related to greater self-reported trait impulsivity [48]. Potentially, our findings may indicate disrupted integration of perceptual information, which could undermine sensory awareness relevant for higher-order decision-making processes, including the inhibition of alcohol use urges [49].

### Asymptotical surprise produces fine-grained modular partitions beyond the resolution limit

In contrast to previous work [19], we identified many brain modules across groups at varying scales, ranging from singleton modules up to modules spanning around 40% of the entire network. This is attributable to the adoption of a more fine-grained community detection approach based on the maximization of Asymptotical Surprise, which, in contrast to many other popular community detection methods, circumvents the resolution limit that constrains the scale of functional modules [20]. However, despite more fine-grained decompositions, we did not observe any differences regarding the quality of modular partitions between groups, thereby replicating previous reports based on resolution-limited community detection [19]. Potentially, alternative metrics may be better suited to reveal mesoscopic alterations in alcohol dependence at the individual-level. In this regard, we incorporated additional aspects of modular organization, such as modular variability and hub disruption, to provide a more comprehensive analysis beyond modular partition quality.

### Increased modular variability in cortical control regions might reflect a neural substrate for relapse risk in alcohol dependence

In the present study, we identified patterns of inter-individual modular variability by comparing the spatial consistency of modular assignments across participants. We found that modular partitions in relapsers were spatially less stable compared to abstainers and controls. Greater variability in modular assignments was especially evident in prefrontal, superior parietal, and temporal regions, aligning with prior findings of pronounced inter-subject variability in heteromodal association areas [33, 50]. In our study, relapsers showed greater modular variability compared to abstainers in the dlPFC, vmPFC, inferior and middle temporal gyri, superior parietal cortex, and amygdala. Such variability may suggest a shift toward a more fragmented and randomized modular organization, where these regions fail to consistently integrate into stable networks, potentially disrupting communication and integration. Notably, the dlPFC, superior parietal, and inferior temporal cortices are central to the frontoparietal network [51], supporting goal-directed behavior and executive control [52, 53], while the vmPFC and amygdala play key roles in emotion regulation [54, 55]. In this context, increased modular variability is unlikely to reflect adaptive network flexibility, but instead may indicate impaired stabilization of regulatory subnetworks at rest. Such instability may undermine reliable top-down control over affective and motivational processes, consistent with impaired control models of addiction [3, 56], thereby contributing to the executive and emotional regulation difficulties frequently seen in alcohol dependence [57, 58], a known relapse risk factor [59–61].

In support of this, our findings showed that greater modular variability increased the hazard of relapse among patients with alcohol dependence. Specifically, each standard deviation increase in modular variability was associated with a 55% higher risk of relapse over the 12-months follow-up period. This effect size is comparable to, or exceeds, those reported for several established clinical predictors of relapse, such as measures of severity, craving, and impulsivity [62], or other brain network-based measures [10]. These predictors typically show modest to moderate associations when considered individually, however, direct comparisons across studies should be interpreted with caution. Taken together, these findings suggest that modular variability captures clinically relevant information beyond traditional risk markers.

Emerging evidence suggests that neuromodulatory approaches could help restore dysfunctional neural circuits. For example, tDCS targeting the dlPFC has been shown to enhance functional connectivity within two large-scale brain networks previously linked to relapse risk over a 4-month follow-up [49, 63]. Importantly, this connectivity increase was accompanied by a substantial reduction in relapse rates [63], highlighting the potential of neuromodulation to normalize mesoscale network disruptions linked to relapse. Of note, modular variability was computed relative to other subjects within the same group. Future studies could instead reference a control group, thereby providing a more normative measure.

### Reward-related brain regions emerge as brain hubs in relapsers

We observed a marked reorganization of functional hubs in prospective relapsers. Hubs denote brain regions that are crucial for inter-module communication, thereby facilitating network integration and efficiency [24]. The negative hub disruption index in relapsers indicates that hub nodes identified in abstainers and controls were less important for inter-module communication in relapsers, and vice versa. Particularly, lower hubness was observed mainly in supplementary motor area, occipital polar cortex and superior temporal cortex – regions pivotal for diverse sensory processing functions [64–66] – suggesting a potential disruption in sensory integration among relapsers. This is consistent with our finding of a fragmentation of the sensory network into a visual, a dorsal somatomotor and a ventral somatomotor component observed in relapsers at the group-level.

Conversely, relapsers exhibited higher hubness primarily in the dorsomedial prefrontal cortex (dmPFC), medial orbitofrontal cortex (mOFC) and subgenual anterior cingulate cortex (sgACC), key regions of the reward network, which are pivotal for the appraisal of subjective value to guide decision-making [67–69]. Previous research has shown stronger alcohol cue-induced activation in mPFC, OFC and ACC in patients with alcohol dependence, being associated with elevated craving and increased relapse risk [70–72]. In the present study, these regions exhibited higher hubness during resting-state when no drug-related cues were present, suggesting that our findings might reflect a baseline vulnerability of reward system dysregulation characterized by an increased intrinsic neural sensitivity to reward, which might predispose individuals to seek alcohol, and consequently increases relapse risk.

Remarkably, abstainers and controls exhibited largely consistent hub architectures, indicated by a hub disruption index near zero. This finding suggests that the central backbone structure of functional brain networks seems to be largely preserved in patients with alcohol dependence that manage to abstain from further alcohol use, corroborating previous findings of a comparable global network topology between these two groups [10].

### Limitations

Several limitations of the present study need to be considered when interpreting the results. Firstly, a relatively modest sample size was obtained within the group of prospective abstainers compared to both relapsers and controls. This imbalance is expected, given that high relapse rates constitute a defining characteristic of alcohol dependence [4]. Nevertheless, future studies should employ larger and more balanced samples to enhance statistical power and sensitivity, thereby improving the detection of group differences between relapsers and abstainers and enabling more robust conclusions regarding neural correlates of relapse vulnerability. Secondly, results from the present study are based on static community detection, wherein modular partitions were extracted from functional connectivity averaged over the entire MRI scanning session. However, brain regions may form transient functional modules that flexibly reconfigure over time, referred to as dynamic modularity [6, 73, 74]. Exploring the temporal dynamics of brain network modularity may provide additional insights into how the brain adapts and reconfigures in alcohol dependence and should therefore be addressed by future work. Thirdly, despite the longitudinal 12-month follow-up assessment of relapse status, group differences in functional brain network organization relied on cross-sectional comparisons. While the observed network characteristics are consistent with a vulnerability-related pattern that appears stable across early abstinence and independent of clinical state measures, the present cross-sectional design does not allow definitive conclusions about trait-like neural markers. Future research should therefore incorporate repeated fMRI assessments to directly examine the temporal stability and evolution of functional brain network organization over time.

## Conclusion

In sum, the present study provides a mesoscopic view on brain network alterations associated with relapse risk in alcohol dependence, thus bridging the gap between whole-brain (macroscale) and nodal (microscale) topology. By employing an unbiased, data-driven community detection approach, we demonstrate an aberrant modular organization in prospective relapsers, marked by fragmentation and reorganization of major functional modules. Moreover, prefrontal reward-related brain regions were more central for inter-module communication in relapsers, emerging as functional brain hubs. Across individuals, functional modules of relapsers exhibited higher spatial variability, particularly in regions associated with behavioral and emotional regulatory processes. Importantly, higher modular variability was associated with time to relapse during follow-up, highlighting the potential relevance of mesoscopic brain network alterations for relapse prediction. Collectively, our results shed light on potential neural substrates of relapse risk in alcohol dependence, which may foster the development of targeted interventions to promote sustained abstinence. Future transdiagnostic studies will be required to determine whether these alterations reflect alcohol-specific mechanisms or a more general vulnerability factor contributing to relapse risk across substance use disorders.

## Supplementary Information

Below is the link to the electronic supplementary material.

Supplementary Material 1

## Data Availability

The raw data are not publicly available due to privacy and ethical restrictions. Derived data may be provided upon reasonable request to the corresponding author. Analysis scripts used in the present study are available at https://github.com/JustinBoehmer/modularity.
