# Supplementary material for: Relapse in alcohol dependence is characterized by disrupted modular brain network organization
Source: Eur Arch Psychiatry Clin Neurosci. 2026 Jan 28;276(4):1733–48. doi: 10.1007/s00406-026-02198-x (PMC13233660; doi:10.1007/s00406-026-02198-x)
Supplement: Supplementary file 1 — Supplementary Material 1 [file 406_2026_2198_MOESM1_ESM.docx]

**SUPPLEMENTARY INFORMATION**

**Relapse in alcohol dependence is characterized by disrupted modular brain network organization**

Justin Böhmer^1,2^; Michael Marxen^3^; Ilya M. Veer^4^; Maria Garbusow^1,5^; Marco Bottino^3^; Ulrich S. Zimmermann^6^; Michael N. Smolka^3^; Andreas Heinz^1,7,8^; Eva Friedel^1^; Christine Heim^2,7,8^; Johann D. Kruschwitz^1,9^*; Henrik Walter^1,7^*

^1^Department of Psychiatry and Psychotherapy CCM, Charité – Universitätsmedizin Berlin, Corporate Member of Freie Universität Berlin, Humboldt-Universität zu Berlin, and Berlin Institute of Health, Berlin, Germany

^2^Institute of Medical Psychology, Charité – Universitätsmedizin Berlin, Corporate Member of Freie Universität Berlin, Humboldt-Universität zu Berlin, and Berlin Institute of Health, Berlin, Germany

^3^Department of Psychiatry and Psychotherapy, Technische Universität Dresden, Dresden, Germany

^4^Department of Developmental Psychology, University of Amsterdam, Amsterdam, the Netherlands

^5^Department of Psychology, MSB Medical School Berlin, Berlin, Germany

^6^Department of Addiction Medicine and Psychotherapy, kbo-Isar-Amper-Klinikum München-Ost, Haar, Germany

^7^German Center for Mental Health, Berlin Potsdam Partner Site, Berlin, Germany

^8^NeuroCure Cluster of Excellence, Berlin, Germany

^9^Department of Psychology, MSB Medical School Berlin, Berlin, Germany

*Shared senior authorship

Contact information of the corresponding author:

Justin Böhmer, Mind and Brain Research Division, Department of Psychiatry and Psychotherapy CCM, Charité – Universitätsmedizin Berlin, Charitéplatz 1, 10117 Berlin, Germany
Email: justin.boehmer@charite.de

**Table of contents**

Extended Methods

Extended Results

Sample characteristics

Table S1. Module labeling based on overlap with canonical brain network templates

Table S2. Differences in modular variability (MV) between REL and CON by regions

Table S3. Differences in modular variability (MV) between REL and ABS by regions

Table S4. Partial correlation between significantly altered mesoscale network metrics in REL and clinical variables of alcohol use

Table S5. Univariate Cox proportional hazards models to investigate the association between altered network metrics in REL and time to relapse

Table S6. Multivariate stratified Cox proportional hazards models to investigate the association between altered network metrics in REL and time to relapse, controlling for covariates

Figure S1. Percolation threshold analyses for all three groups

Figure S2. Scatterplots illustrating the associations between percolation-based network density and mesoscale network metrics

Figure S3. Individual distributions of mesoscale network metrics

Figure S4. Robustness analysis using proportional thresholding

**Extended Methods**

*Permutation procedure to test for differences in modular decomposition*

We computed the correspondence between network partitions using the normalized mutual information index (NMI [1]), accounting for differences in the number of modules between groups [2]. To test for statistical significance between groups, we conducted a permutation procedure introduced by Alexander-Bloch et al. [3] based on individual-level community partitions. Briefly, if deviations in modular assignments are attributable to group membership, then the mean similarity between all possible pairs of participants within an experimental group should be higher than the mean similarity of pairs of participants from random groups [3]. As the underlying distribution of the group means similarity is unknown, we generated a null distribution by permuting the group labels 10,000 times. The number of instances in which the within-group similarity of the empirical groups exceeded the within-group similarity of permuted groups, divided by the number of permutations, indicated the *p*-value. Modular partitions were considered significantly different between groups if *p* < .05.

*Modular variability*

We investigated the spatial variability of modular partitions across subjects using the modular variability (*MV*) metric [4]. *MV* is a measure defined for a subject pair and for a single node, indicating the overlap of the modules to which the node belongs, normalized by the size of the two modules. For each subject, we calculated the mean *MV* between this subject‘s modular partition and the partitions from all other subjects of the same group. *MV* ranges between 0 and 1, with lower values indicating that a particular node is consistently assigned to the same module across subjects while larger *MV* scores indicate higher variability of module affiliations. The mean *MV* across subjects of a given group indicates the spatial variability of modular partitions within this group. We compared the mean *MV* value for each region of the brain and the mean across all brain regions between groups using nonparametric permutation tests (10,000 permutations), controlling for age, gender, site, years of education, mean framewise displacement and the percolation-based sparsity threshold (Bonferroni-corrected).

*Identification of hub structures and hub disruption index*

To identify hub structures within the network, we calculated the normalized participation coefficient (*PC_norm_*), reflecting the extent to which a node is connected to nodes in other modules. *PC_norm_* provides a measure of inter-module connectivity independent of the size and connectedness of the modules by incorporating a network randomization approach [5]. We calculated *PC_norm_* based on 1,000 network randomizations. As the computation of *PC_norm_* depends on the underlying network density, we employed proportional sparsity thresholds, ensuring equal network density across subjects. Specifically, we computed *PC_norm_* across the range of 5% to 50% edge density, incrementing in 5% steps. Corresponding modular partitions were derived at each threshold using PACO. *PC_norm_* values were then averaged across thresholds for each subject.

To assess differences in hub assignments across groups, we computed the hub disruption index *κ* [6]. In accordance with previous studies [7,8], *κ* corresponds to the slope of the linear regression line fitted to the *PC_norm_* values of each brain region from a reference group versus the difference in *PC_norm_* of each brain region between the reference group and the subject/group under study. For instance, the hub disruption index for the REL group corresponds to the slope of the linear regression line fitted to the *PC_norm_* of the CON group (i.e., the reference group) versus the difference in *PC_norm_* between CON and REL. For inference statistical testing, *κ* was estimated for each subject by contrasting the individual-level *PC_norm_* with the group-averaged *PC_norm_* of CON and comparing it between groups. All group comparisons were carried out using permutation tests (10,000 permutations) controlling for age, sex, site, years of education and mean framewise displacement (Bonferroni-corrected).

**Extended Results**

*Robustness and sensitivity analyses*

(1) Reliability of mesoscale network metrics

We assessed test–retest reliability of the mesoscale network metrics using a split-half procedure based on odd–even volume partitioning. The metrics showed good to excellent reliability, with Spearman–Brown–corrected coefficients of *r_SB_* = 0.87 for modular variability, *r_SB_* = 0.92 for modular partition quality, *r_SB_* = 0.75 for the number of modules and for mean module size, *r_SB_* = 0.74 for module radius, and *r_SB_* = 0.82 for the normalized participation coefficient.

(2) Adjustment for clinical covariates in alcohol-dependent groups

We performed sensitivity analyses by recomputing the permutation-based pairwise comparison between REL and ABS while including relevant clinical covariates (addiction severity, craving, number of abstinence days, smoking), together with the original set of covariates (age, sex, site, education, mean FD). Additional adjustment for addiction severity (*p* = .004), baseline craving (*p* = .007), number of abstinent days prior to MRI (*p* = .006), and nicotine use (*p* = .003) did not alter the significance or direction of the observed difference in modular variability between REL and ABS, indicating that the effect is robust to these potential confounders.

Moreover, we assessed associations between modular variability and baseline clinical state measures. Across participants with alcohol dependence, modular variability showed no significant correlations with baseline craving (*r* = 0.07, *p* = .511), addiction severity (*r* = –0.12, *p* = .253), abstinence duration prior to MRI (*r* = –0.07, *p* = .506), or nicotine use (*r* = 0.16, *p* = .136).

(3) Site-stratified analyses

For modular variability (MV) and normalized participation coefficient (*PC_norm_*), differences between REL and CON replicated within both Berlin (*p_MV_* = .007, *p_PCnorm_* = .030, Bonferroni-corrected) and Dresden (*p_MV_* = .012, *p_PCnorm_* = .027, Bonferroni-corrected), with same effect directions and comparable effect sizes, supporting the robustness of these findings across sites. For percolation-based network density and the hub disruption index, the direction of REL-related differences was consistent across both sites. However, significance was reached only in Berlin (*p_Density_* = .047, *p_HDI_* = .019, Bonferroni-corrected) but not in Dresden (*p_Density_* = .214, *p_HDI_* = .338). We attribute this to reduced statistical power in the smaller site (Dresden: *n* = 77) compared to the larger site (Berlin: *n* = 93) rather than to a site-driven effect, as no evidence of effect reversals or site-specific divergence was observed.

In addition, we performed ComBat harmonization of the functional connectivity (FC) matrices (covariates: age, sex, education, group, mean FD) to evaluate potential scanner- or site-related biases. Harmonization produced negligible changes in FC, with an almost perfect mean correlation between harmonized and original FC across participants (*M* = 0.9991, *SD* = 0.0004). Importantly, these correlations did not differ between groups (*F*(2,167) = 0.32, *p* = .725), indicating an absence of group-specific or site-specific harmonization effects.

(4) Consensus-based modular partitions

We conducted a sensitivity analysis to assess the stability and reproducibility of the community detection results. Specifically, we computed consensus-based modular partitions across 5,000 PACO runs and compared these to the modular partition with the highest Asymptotical Surprise (SA) value, which we originally selected. The similarity between the consensus-based and highest-SA partitions, quantified using normalized mutual information (*NMI*), was high across all groups (CON: *NMI* = 0.92; ABS: *NMI* = 0.92; REL: *NMI* = 0.91), indicating that both approaches converged on a nearly identical modular structure.

To further evaluate stability, we examined the similarity among the top 100 partitions with the highest SA values, and additionally computed similarity across all 5,000 partitions and between the partitions with the highest and the lowest SA value. The mean similarity among the top 100 partitions (*NMI_top100_* = 0.92) was only slightly higher than the mean similarity across all 5,000 runs (*NMI_all5000_* = 0.88), and even the similarity between the highest- and lowest-SA partitions remained high (*NMI_highest-vs-lowest_* = 0.85). Similar patterns were observed for the REL group (*NMI_top100_* = 0.91, *NMI_all5000_* = 0.87, *NMI_highest-vs-lowest_* = 0.83) and the ABS group (*NMI_top100_* = 0.92, *NMI_all5000_* = 0.85, *NMI_highest-vs-lowest_* = 0.78). Together, these findings demonstrate that PACO consistently converged on highly similar solutions, with only minimal variability across runs.

(5) Alternative brain parcellation scheme

We performed a sensitivity analysis using an alternative brain parcellation. We selected the Shen atlas [17], as it includes both cortical and subcortical regions, similar to the Brainnetome Atlas. Using the Shen parcellation, percolation-based network density remained significantly lower in REL compared to CON (*p* = .035), consistent with our original findings. Likewise, REL again showed higher modular variability relative to CON (*p* = .004). The comparison between REL and ABS showed the same effect direction but did not reach significance after Bonferroni correction (*p* = .083). For the participation coefficient, we observed the same pattern as with the original parcellation, with REL showing lower values compared to CON (*p* = .003). As with the primary parcellation, no significant group differences were observed for modular partition quality (*p* = .101), number of modules (*p* = .374), mean module size (*p* = .976), or module radius (*p* = .432). Overall, these results largely replicate the main findings and support their robustness across parcellation schemes.

**Table S1**. Module labeling. Spatial correlation between the seven largest modules from the group-level community partition in the CON group and five large-scale canonical brain network templates. Module labels were assigned based on the largest spatial correlation and the core brain regions constituting the module.


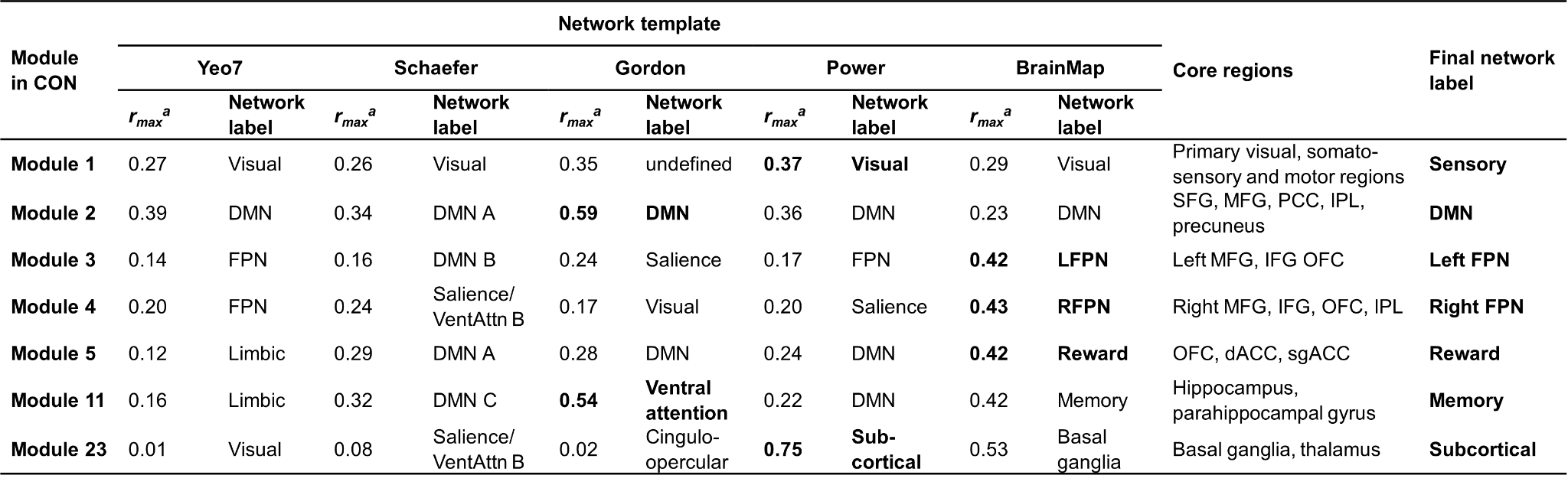


^a^Largest spatial correlation [10] between the respective module in CON and all networks from the respective canonical network template. Bold values indicate the largest spatial correlation across canonical network templates. Network templates: Yeo7 [11], Schaefer [12], Gordon [13], Power [14], BrainMap [15]. Abbreviations: CON = Control, DMN = Default Mode Network, FPN = Fronto-Parietal Network, VentAttn = Ventral Attention Network, SFG = superior frontal gyrus, MFG = middle frontal gyrus, PCC = posterior cingulate cortex, IPL = inferior parietal lobule, IFG = inferior frontal gyrus, OFC = orbitofrontal cortex, dACC = dorsal anterior cingulate cortex, sgACC= subgenual anterior cingulate cortex.

**Table S2**. Differences in modular variability (MV) between REL and CON by regions.
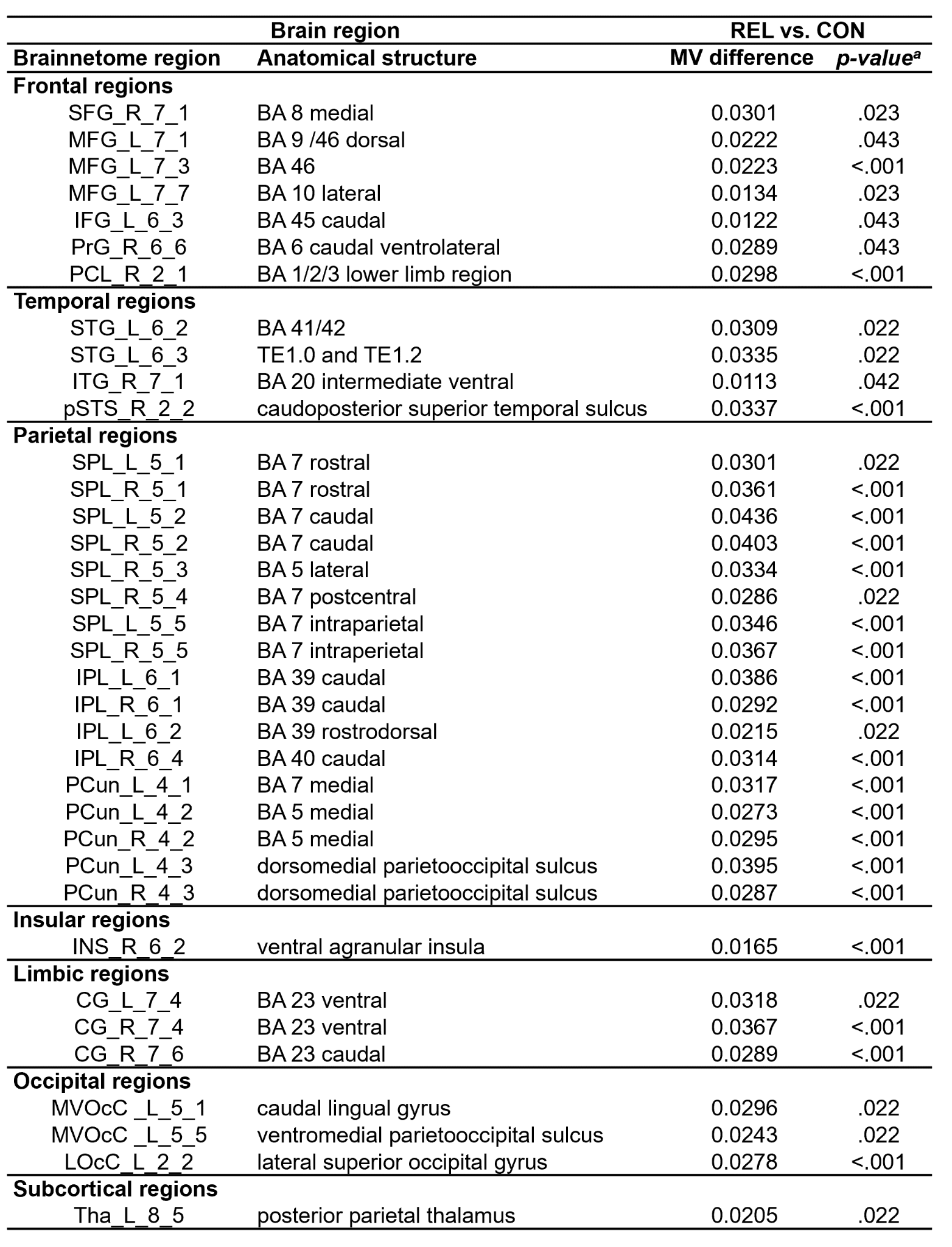


^a^*p-*value based on 10,000 permutations (Bonferroni corrected). Brain region labels based on Brainnetome Atlas [16]. Abbreviations: REL = Relapsing AD patients, CON = controls, MV = modular variability, BA = Brodmann area, SFG = superior frontal gyrus, MFG = middle frontal gyrus, IFG = inferior frontal gyrus, PrG = precentral gyrus, PCL = paracentral lobule, STG = superior temporal gyrus, ITG = inferior temporal gyrus, pSTS = posterior superior temporal sulcus, SPL = superior parietal lobule, IPL = inferior parietal lobule, PCun = precuneus, INS = insula, CG = cingulate gyrus, MVOcC = medioventral occipital cortex, LOcC = lateral occipital cortex, Tha = thalamus, R = right, L = left.

**Table S3**. Differences in modular variability (MV) between REL and ABS by regions.
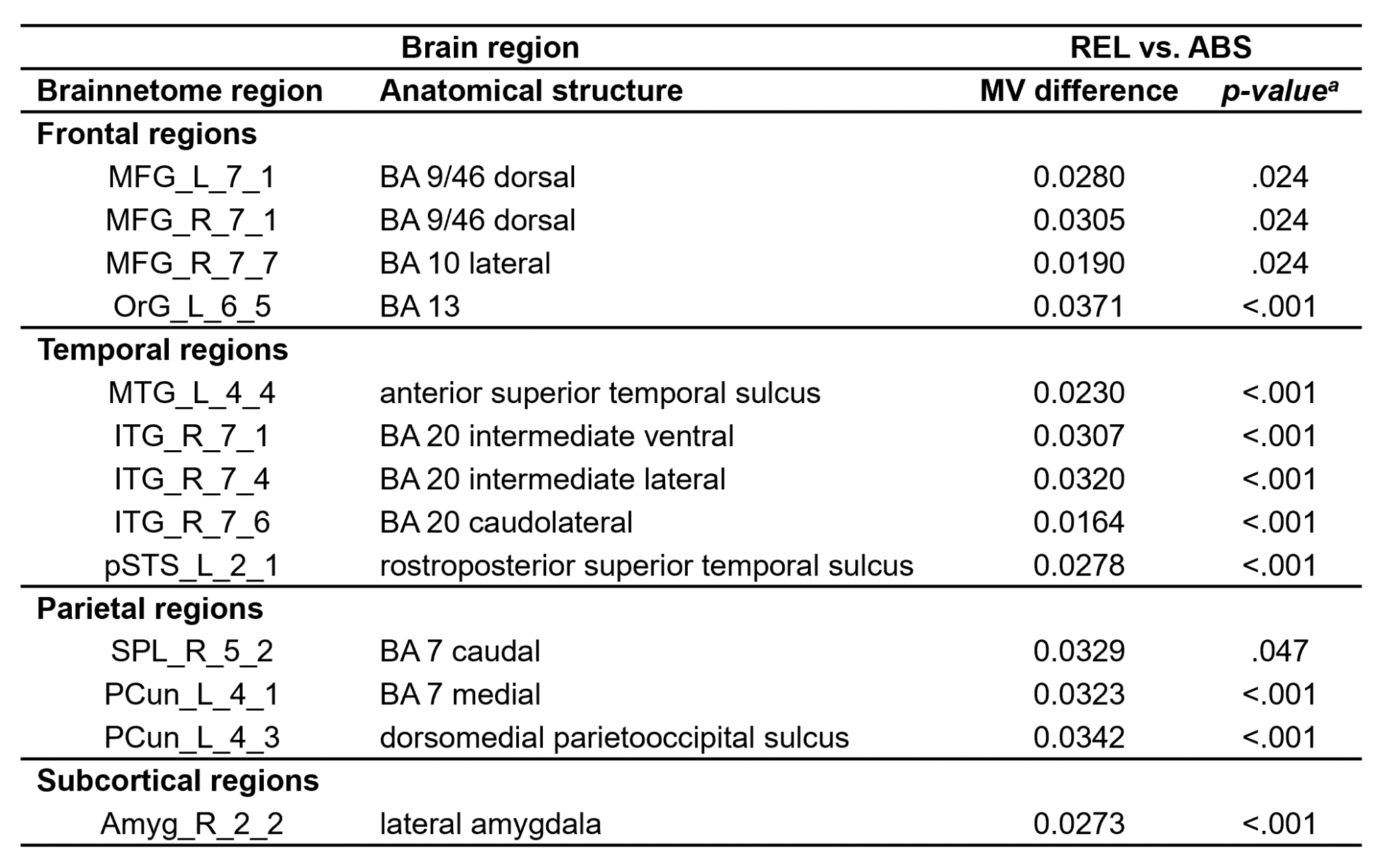


^a^*p-*value based on 10,000 permutations (Bonferroni corrected). Brain region labels based on Brainnetome Atlas [16]. Abbreviations: REL = Relapsing AD patients, ABS = Abstaining AD patients, MV = modular variability, BA = Brodmann area, MFG = middle frontal gyrus, OrG = orbitofrontal gyrus, MTG = middle temporal gyrus, ITG = inferior temporal gyrus, pSTS = posterior superior temporal sulcus, SPL = superior parietal lobule, PCun = precuneus, Amyg = amygdala, Tha = thalamus, R = right, L = left.

**Table S4**. Partial correlation between significantly altered mesoscale network metrics in REL and clinical variables of alcohol use.

| **Network metric** | **Clinical variable** | | | | | | | | | | | | | | | | | | | |  |
| --- | --- | --- | --- | --- | --- | --- | --- | --- | --- | --- | --- | --- | --- | --- | --- | --- | --- | --- | --- | --- | --- |
|  | **CIDI_D1** | | **CIDI_D2** | | **CIDI_D3** | | **CIDI_D4** | | **CIDI_D5** | | **CIDI_D6** | | **ADS** | | **OCDS** | | **TLFB** | | **Abst.Days** | | |
|  | *r* | *p* | *r* | *p* | *r* | *p* | *r* | *p* | *r* | *p* | *r* | *p* | *r* | *p* | *r* | *p* | *r* | *p* | *r* | *p* | |
| Hub disruption index | -0.23 | .114 | -0.15 | .295 | -0.23 | .091 | -0.20 | .147 | -0.15 | .267 | -0.16 | .237 | -0.20 | .141 | 0.05 | .737 | -0.05 | .724 | -0.06 | .674 | |
| Percolation-based density | -0.22 | .134 | 0.05 | .727 | 0.06 | .645 | -0.06 | .661 | -0.08 | .546 | -0.12 | .371 | -0.09 | .515 | 0.21 | .144 | 0.00 | .982 | -0.03 | .831 | |
| MV_global | 0.24 | .096 | -0.03 | .842 | 0.14 | .317 | 0.26 | .059 | 0.18 | .198 | 0.16 | .245 | 0.04 | .767 | 0.07 | .625 | -0.06 | .681 | 0.00 | .994 | |
| PC_norm_ | -0.01 | .928 | 0.11 | .411 | -0.03 | .822 | -0.04 | .789 | 0.16 | .263 | 0.01 | .941 | 0.13 | .353 | -0.07 | .615 | 0.13 | .352 | -0.14 | .305 | |
| MV_local: SFG_R_7_1 | 0.19 | .180 | -0.18 | .197 | -0.02 | .893 | 0.27 | .048 | 0.06 | .670 | 0.10 | .485 | -0.08 | .543 | -0.10 | .473 | 0.00 | .997 | -0.07 | .631 | |
| MV_local: MFG_L_7_1 | 0.08 | .605 | -0.19 | .180 | -0.02 | .863 | 0.05 | .731 | 0.00 | .996 | 0.12 | .402 | -0.20 | .139 | 0.00 | .988 | -0.18 | .181 | 0.13 | .367 | |
| MV_local: MFG_L_7_3 | 0.18 | .220 | -0.03 | .834 | -0.04 | .792 | 0.25 | .065 | 0.24 | .085 | 0.21 | .130 | -0.02 | .869 | 0.30 | .031 | -0.17 | .221 | 0.11 | .422 | |
| MV_local: MFG_L_7_7 | 0.14 | .346 | -0.10 | .474 | 0.09 | .538 | 0.34 | .012 | 0.23 | .091 | 0.28 | .038 | 0.02 | .901 | 0.12 | .410 | -0.09 | .497 | 0.03 | .851 | |
| MV_local: IFG_L_6_3 | 0.03 | .856 | 0.10 | .493 | 0.03 | .853 | 0.13 | .348 | -0.02 | .906 | 0.00 | .983 | 0.21 | .135 | -0.14 | .338 | -0.07 | .607 | 0.01 | .916 | |
| MV_local: PrG_R_6_6 | 0.25 | .085 | 0.02 | .868 | 0.16 | .235 | 0.13 | .344 | 0.00 | .992 | 0.02 | .886 | 0.11 | .420 | 0.13 | .352 | -0.13 | .357 | 0.01 | .939 | |
| MV_local: PCL_R_2_1 | 0.15 | .305 | 0.06 | .681 | 0.14 | .300 | 0.18 | .181 | 0.20 | .148 | 0.14 | .303 | 0.05 | .721 | 0.01 | .939 | -0.09 | .538 | 0.01 | .924 | |
| MV_local: STG_L_6_2 | 0.18 | .208 | 0.19 | .176 | 0.23 | .089 | -0.05 | .744 | 0.06 | .670 | -0.03 | .846 | -0.09 | .531 | 0.05 | .750 | -0.09 | .495 | -0.12 | .395 | |
| MV_local: STG_L_6_3 | 0.26 | .075 | 0.14 | .303 | 0.20 | .154 | 0.08 | .588 | 0.09 | .496 | 0.11 | .426 | 0.14 | .309 | 0.16 | .263 | -0.22 | .117 | -0.10 | .480 | |
| MV_local: ITG_R_7_1 | 0.02 | .911 | 0.08 | .551 | 0.10 | .485 | 0.30 | .030 | 0.19 | .159 | 0.19 | .165 | 0.02 | .875 | 0.33 | .017 | -0.03 | .842 | -0.05 | .695 | |
| MV_local: pSTS_R_2_2 | 0.25 | .083 | 0.07 | .602 | 0.10 | .491 | 0.19 | .165 | 0.08 | .569 | 0.05 | .700 | 0.08 | .584 | 0.17 | .221 | 0.01 | .931 | 0.09 | .523 | |
| MV_local: SPL_L_5_1 | 0.08 | .579 | 0.03 | .829 | 0.16 | .236 | 0.28 | .037 | 0.11 | .435 | 0.20 | .153 | -0.04 | .768 | -0.09 | .526 | -0.07 | .606 | -0.04 | .751 | |
| MV_local: SPL_R_5_1 | 0.18 | .210 | 0.01 | .934 | 0.18 | .187 | 0.22 | .113 | 0.14 | .307 | 0.24 | .084 | 0.03 | .822 | -0.02 | .900 | -0.07 | .609 | -0.02 | .862 | |
| MV_local: SPL_L_5_2 | 0.09 | .546 | 0.03 | .845 | 0.10 | .476 | 0.24 | .086 | 0.19 | .173 | 0.29 | .031 | 0.11 | .443 | -0.09 | .516 | -0.15 | .289 | 0.00 | .999 | |
| MV_local: SPL_R_5_2 | 0.11 | .453 | -0.06 | .666 | 0.03 | .856 | 0.22 | .113 | 0.17 | .207 | 0.22 | .103 | 0.18 | .184 | -0.09 | .539 | -0.10 | .484 | -0.01 | .953 | |
| MV_local: SPL_R_5_3 | 0.12 | .417 | 0.08 | .549 | 0.18 | .191 | 0.24 | .082 | 0.28 | .040 | 0.23 | .089 | 0.01 | .962 | -0.02 | .863 | -0.11 | .436 | -0.02 | .904 | |
| MV_local: SPL_R_5_4 | 0.09 | .542 | -0.03 | .816 | 0.12 | .369 | 0.15 | .267 | 0.08 | .587 | 0.09 | .527 | 0.11 | .448 | 0.00 | .977 | -0.07 | .621 | -0.02 | .877 | |
| MV_local: SPL_L_5_5 | 0.17 | .249 | -0.01 | .917 | 0.05 | .731 | 0.29 | .034 | 0.36 | .008 | 0.30 | .026 | -0.10 | .474 | -0.14 | .339 | -0.10 | .475 | -0.01 | .967 | |
| MV_local: SPL_R_5_5 | 0.17 | .243 | -0.01 | .925 | 0.11 | .427 | 0.33 | .015 | 0.29 | .036 | 0.31 | .024 | 0.01 | .967 | -0.11 | .447 | -0.06 | .654 | -0.06 | .664 | |
| MV_local: IPL_L_6_1 | 0.25 | .078 | 0.09 | .517 | 0.20 | .151 | 0.16 | .242 | 0.18 | .199 | 0.20 | .142 | 0.05 | .700 | -0.02 | .876 | -0.08 | .545 | 0.06 | .641 | |
| MV_local: IPL_R_6_1 | 0.13 | .387 | 0.11 | .439 | 0.16 | .239 | 0.31 | .022 | 0.21 | .131 | 0.25 | .074 | 0.12 | .373 | 0.13 | .365 | -0.03 | .818 | -0.04 | .753 | |
| MV_local: IPL_L_6_2 | 0.06 | .687 | -0.20 | .152 | -0.04 | .782 | 0.30 | .025 | 0.14 | .324 | 0.22 | .115 | 0.09 | .511 | 0.11 | .425 | -0.11 | .422 | -0.01 | .919 | |
| MV_local: IPL_R_6_4 | 0.01 | .972 | -0.18 | .195 | -0.11 | .441 | 0.06 | .684 | 0.04 | .761 | -0.02 | .881 | -0.27 | .050 | -0.05 | .699 | 0.04 | .774 | -0.03 | .848 | |
| MV_local: PCun_L_4_1 | 0.06 | .693 | -0.07 | .592 | -0.06 | .673 | 0.29 | .035 | 0.36 | .008 | 0.38 | .005 | 0.09 | .500 | -0.09 | .504 | -0.21 | .127 | 0.02 | .886 | |
| MV_local: PCun_L_4_2 | 0.11 | .441 | -0.06 | .678 | 0.12 | .392 | 0.22 | .107 | 0.13 | .338 | 0.19 | .170 | 0.08 | .543 | -0.01 | .952 | -0.14 | .326 | -0.06 | .656 | |
| MV_local: PCun_R_4_2 | 0.12 | .415 | -0.06 | .669 | 0.12 | .386 | 0.23 | .099 | 0.13 | .331 | 0.19 | .163 | 0.09 | .514 | -0.01 | .968 | -0.14 | .325 | -0.07 | .628 | |
| MV_local: PCun_L_4_3 | 0.03 | .843 | 0.04 | .752 | -0.01 | .933 | 0.15 | .284 | 0.25 | .066 | 0.29 | .035 | 0.01 | .953 | -0.06 | .673 | -0.06 | .663 | 0.09 | .507 | |
| MV_local: PCun_R_4_3 | 0.16 | .265 | 0.09 | .502 | 0.07 | .611 | 0.22 | .103 | 0.29 | .035 | 0.33 | .016 | 0.14 | .315 | 0.05 | .721 | -0.10 | .460 | 0.05 | .694 | |
| MV_local: INS_R_6_2 | 0.10 | .497 | 0.02 | .859 | 0.02 | .873 | 0.16 | .248 | 0.03 | .846 | 0.10 | .458 | -0.04 | .759 | -0.12 | .401 | 0.07 | .633 | 0.00 | .997 | |
| MV_local: CG_L_7_4 | 0.12 | .422 | 0.10 | .454 | 0.19 | .174 | 0.02 | .898 | 0.06 | .691 | 0.10 | .451 | 0.00 | .978 | 0.14 | .306 | 0.03 | .842 | 0.09 | .500 | |
| MV_local: CG_R_7_4 | 0.08 | .602 | 0.09 | .510 | 0.15 | .272 | 0.10 | .459 | 0.18 | .193 | 0.22 | .118 | 0.09 | .527 | 0.04 | .774 | -0.13 | .344 | 0.00 | .974 | |
| MV_local: CG_R_7_6 | 0.13 | .371 | -0.02 | .909 | 0.08 | .550 | 0.24 | .079 | 0.25 | .067 | 0.21 | .125 | 0.01 | .952 | 0.10 | .477 | -0.15 | .287 | -0.11 | .431 | |
| MV_local: MVOcC _L_5_1 | 0.14 | .349 | 0.13 | .336 | 0.02 | .912 | 0.00 | .974 | 0.11 | .431 | 0.10 | .466 | 0.09 | .529 | 0.02 | .909 | -0.04 | .801 | 0.00 | .980 | |
| MV_local: MVOcC _L_5_5 | 0.24 | .092 | 0.06 | .683 | 0.16 | .241 | 0.17 | .231 | 0.20 | .148 | 0.15 | .292 | 0.00 | .982 | 0.12 | .400 | -0.03 | .831 | 0.02 | .863 | |
| MV_local: LOcC_L_2_2 | 0.18 | .210 | 0.02 | .903 | 0.09 | .511 | 0.11 | .408 | 0.19 | .176 | 0.12 | .373 | 0.00 | .979 | -0.01 | .956 | -0.05 | .732 | 0.06 | .674 | |
| MV_local: Tha_L_8_5 | 0.06 | .669 | 0.00 | .995 | 0.07 | .601 | 0.11 | .417 | 0.24 | .078 | 0.30 | .030 | 0.04 | .794 | 0.44 | **.001*** | -0.13 | .347 | -0.12 | .406 | |

*p-value significant after Bonferroni correction, correcting for the number of significant mesoscale network metrics (p = .05 / 40 = .00125). Brain region labels based on Brainnetome Atlas [16]. Abbreviations: REL = relapsing alcohol-dependent patients, CIDI_D1 = Age of first drink (based on CIDI), CIDI_D2 = Age of first time drunk (based on CIDI), CIDI_D3 = Age of first binge-drinking episode (based on CIDI), CIDI_D4 = Alcohol consumption per day in g (based on CIDI), CIDI_D5 = Alcohol consumption per occasion in g (based on CIDI), CIDI_D6 = Alcohol consumption per binge-drinking episode in g (based on CIDI), ADS = Alcohol Dependence Scale, OCDS = Obsessive Compulsive Drinking Scale, TLFB = Timeline-Follow-Back, Abst.Days = days of alcohol abstinence prior to MRI scan, MV = modular variability, PC_norm_ = Normalized participation coefficient, SFG = superior frontal gyrus, MFG = middle frontal gyrus, IFG = inferior frontal gyrus, PrG = precentral gyrus, PCL = paracentral lobule, STG = superior temporal gyrus, ITG = inferior temporal gyrus, pSTS = posterior superior temporal sulcus, SPL = superior parietal lobule, IPL = inferior parietal lobule, PCun = precuneus, INS = insula, CG = cingulate gyrus, MVOcC = medioventral occipital cortex, LOcC = lateral occipital cortex, Tha = thalamus, R = right, L = left.

**Table S5**. Univariate Cox proportional hazards models to investigate the association between altered network metrics in REL and time to relapse.

^a^Network metrics significantly altered in REL. ^b^Proportional hazards assumption tested based on Schoenfeld residuals. *Significant after Bonferroni correction, correcting for the number of tested network metrics (*α* = 0.05 / 4 = 0.0125). Abbreviations: PH = proportional hazards, HR = hazards ratio, 𝛘² = Chi-Square test statistic, 95% CI = 95% confidence interval.

| **Network metric^a^** | **Cox proportional hazards models (univariate)** | | | | | | | | | |
| --- | --- | --- | --- | --- | --- | --- | --- | --- | --- | --- |
|  | **Model fit statistics** | | | | **PH assumption test^b^** | | **Effect estimates** | | |  |
|  | *N / No. of events* | *𝛘^2(^df)* | *Harrell’s C* | *p* | *𝛘^2(^df)* | *p* | *HR* | *95% CI* | *p* |  |
| Percolation-based network density | 87 / 59 | 0.35(1) | 0.54 | .553 | 0.02(1) | .883 | 0.91 | 0.68 – 1.23 | .556 |  |
| Modular variability (MV) | 87 / 59 | 10.91(1) | 0.63 | < .001* | 0.08(1) | .778 | 1.55 | 1.19 – 2.02 | .001 |  |
| Participation coefficient (*PC_norm_*) | 87 / 59 | 1.88(1) | 0.57 | .170 | 0.86(1) | .355 | 0.85 | 0.67 – 1.07 | .168 |  |
| Hub disruption index (*κ*) | 87 / 59 | 1.51(1) | 0.55 | .219 | 0.41(1) | .521 | 0.84 | 0.63 – 1.11 | .219 |  |

**Table S6**. Multivariate stratified Cox proportional hazards models to investigate the association between altered network metrics in REL and time to relapse, controlling for covariates.

^a^Stratified Cox proportional hazards models with “site” as stratification variable due to violation of the proportional hazards assumption (*𝛘^2(^df)* = 11.52, *p* < .001). ^b^Proportional hazards assumption tested based on Schoenfeld residuals. Abbreviations: PH = proportional hazards, HR = hazards ratio, 𝛘² = Chi-Square test statistic, 95% CI = 95% confidence interval, FD = framewise displacement.

| **Variable** | **Stratified Cox proportional hazards models (multivariate)^a^** | | | | | | | | | |
| --- | --- | --- | --- | --- | --- | --- | --- | --- | --- | --- |
|  | **Model fit statistics** | | | | **PH assumption test^b^** | | **Effect estimates** | | |  |
|  | *N / No. of events* | *𝛘^2(^df)* | *Harrell’s C* | *p* | *𝛘^2(^df)* | *p* | *HR* | *95% CI* | *p* |  |
| Overall model | 87 / 59 | 11.85(5) | 0.63 | .037 | 5.22(5) | .389 | - | - | - |  |
| Age | - | - | - | - | 0.62(1) | .431 | 0.94 | 0.73 – 1.23 | .670 |  |
| Sex | - | - | - | - | 0.62(1) | .432 | 1.64 | 0.69 – 3.92 | .265 |  |
| Education | - | - | - | - | 3.79(1) | .052 | 0.95 | 0.70 – 1.29 | .736 |  |
| Mean FD | - | - | - | - | 0.02(1) | .880 | 1.02 | 0.79 – 1.31 | .882 |  |
| Modular variability (MV) | - | - | - | - | 0.06(1) | .802 | 1.56 | 1.18 – 2.05 | .002 |  |


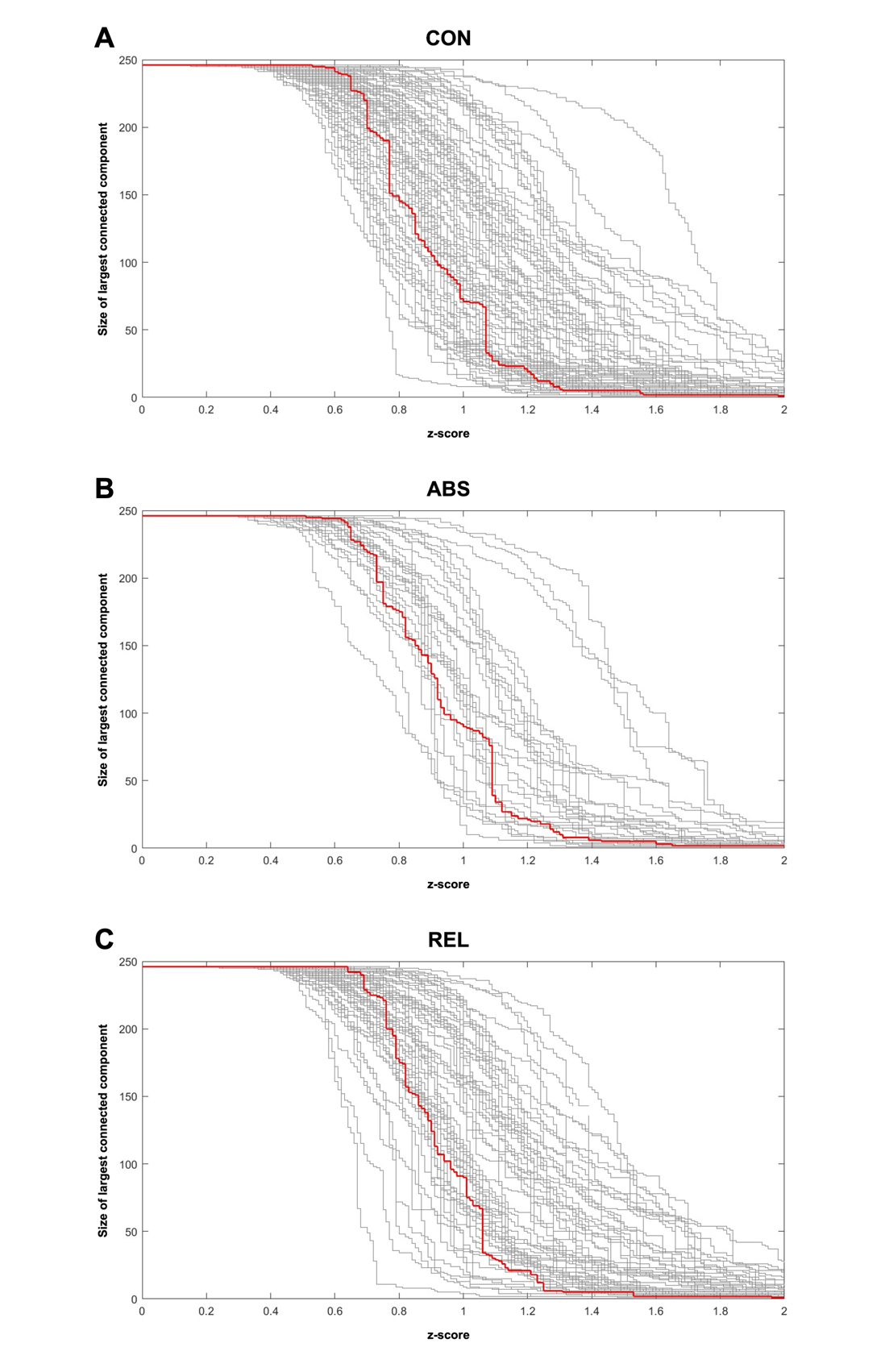


**Figure S1**. Percolation threshold analyses in **A)** controls (CON), **B)** abstaining AD patients (ABS) and **C)** relapsing AD patients (REL). Percolation curves indicate the size of the largest connected component upon iterative edge removal. Grey lines correspond to percolation curves from individual subjects while red lines represent the percolation curve based on the group-averaged matrices. For modularity analyses, all individual- and group-level connectivity matrices were thresholded at the percolation point, i.e., the z-score at which the largest connected component starts breaking into smaller subnetworks.


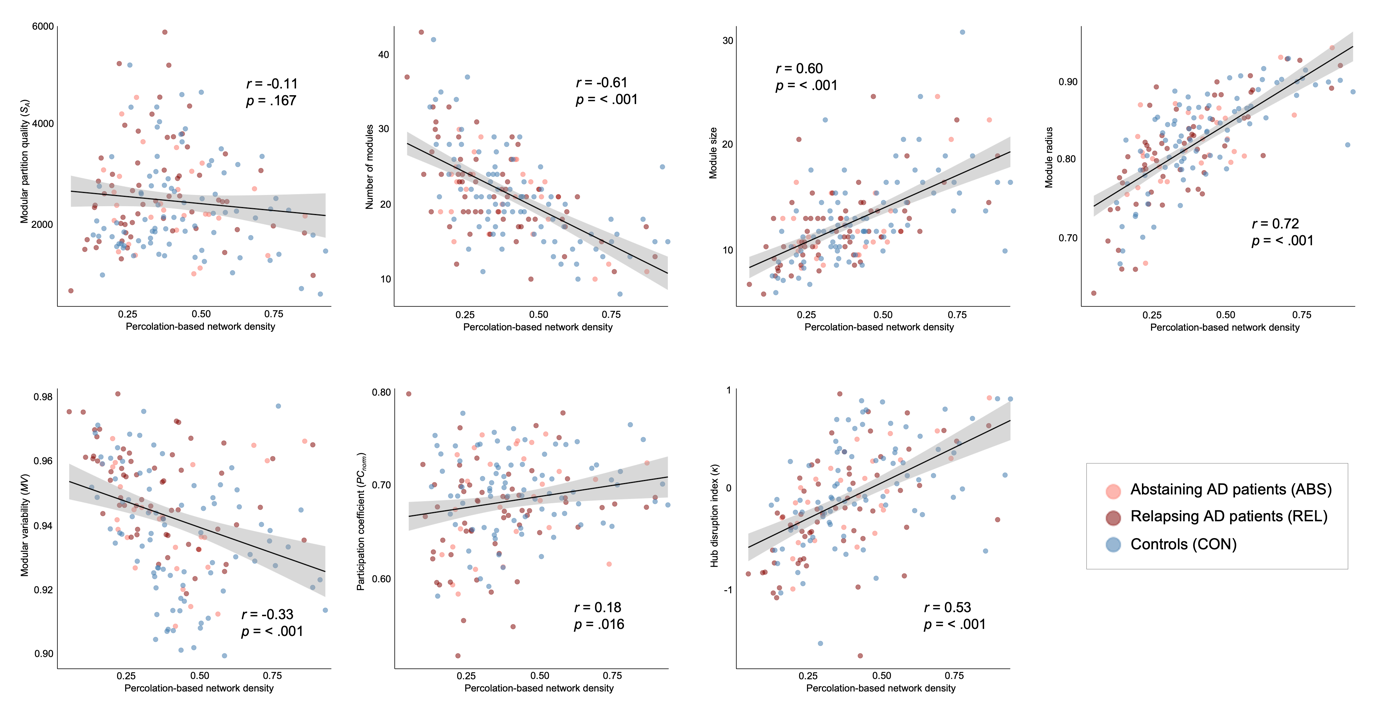


**Figure S2.**Scatterplots illustrating the associations between percolation-based network density and mesoscale network metrics. Points represent individual participants (ABS = abstinent alcohol-dependent patients, REL = relapsing alcohol-dependent patients, CON = controls). Regression lines with 95% confidence intervals are shown.


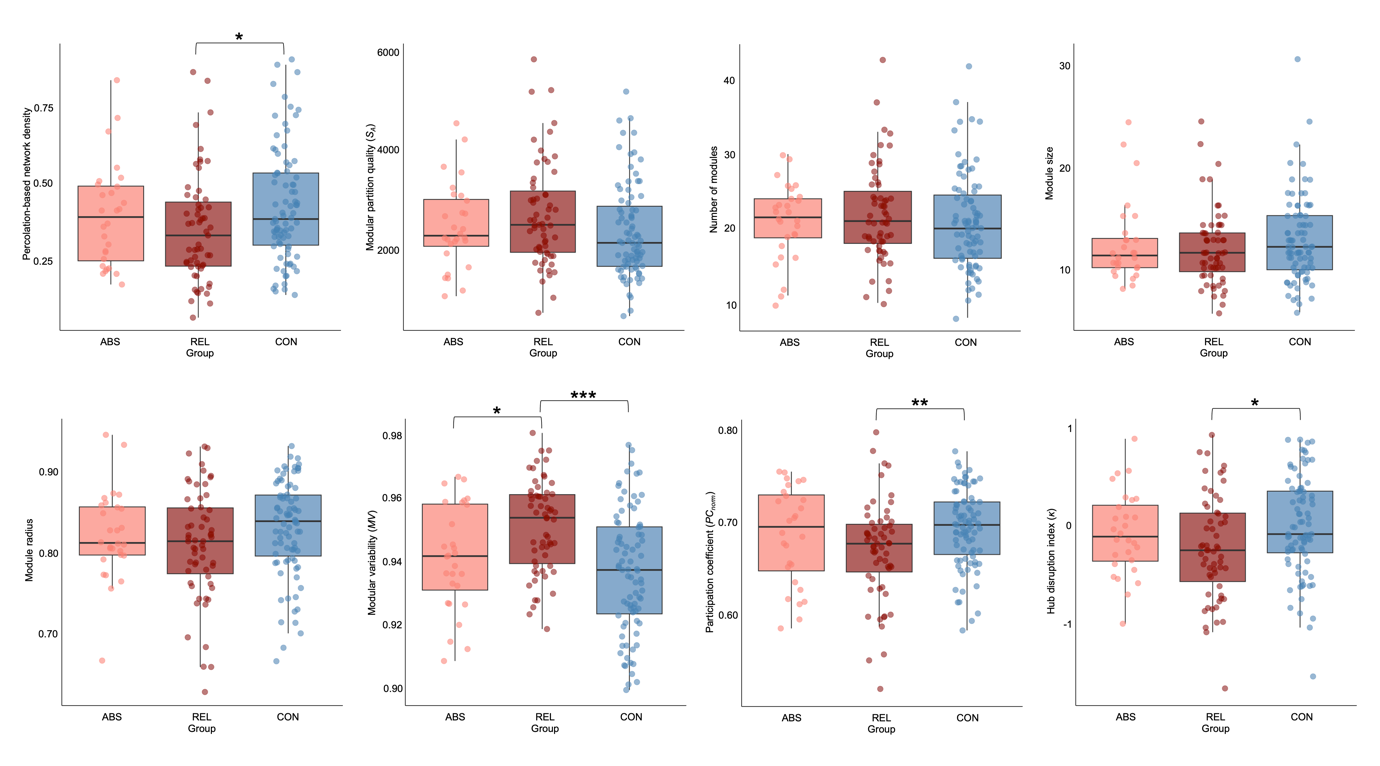


**Figure S3.** Individual distributions of mesoscale network metrics across abstinent alcohol-dependent patients (ABS), relapsing patients (REL), and healthy controls (CON). Boxplots show median and interquartile range; dots represent individual participants. Significance levels: * *p* < .05, ** *p* < .01, *** *p* < .001.


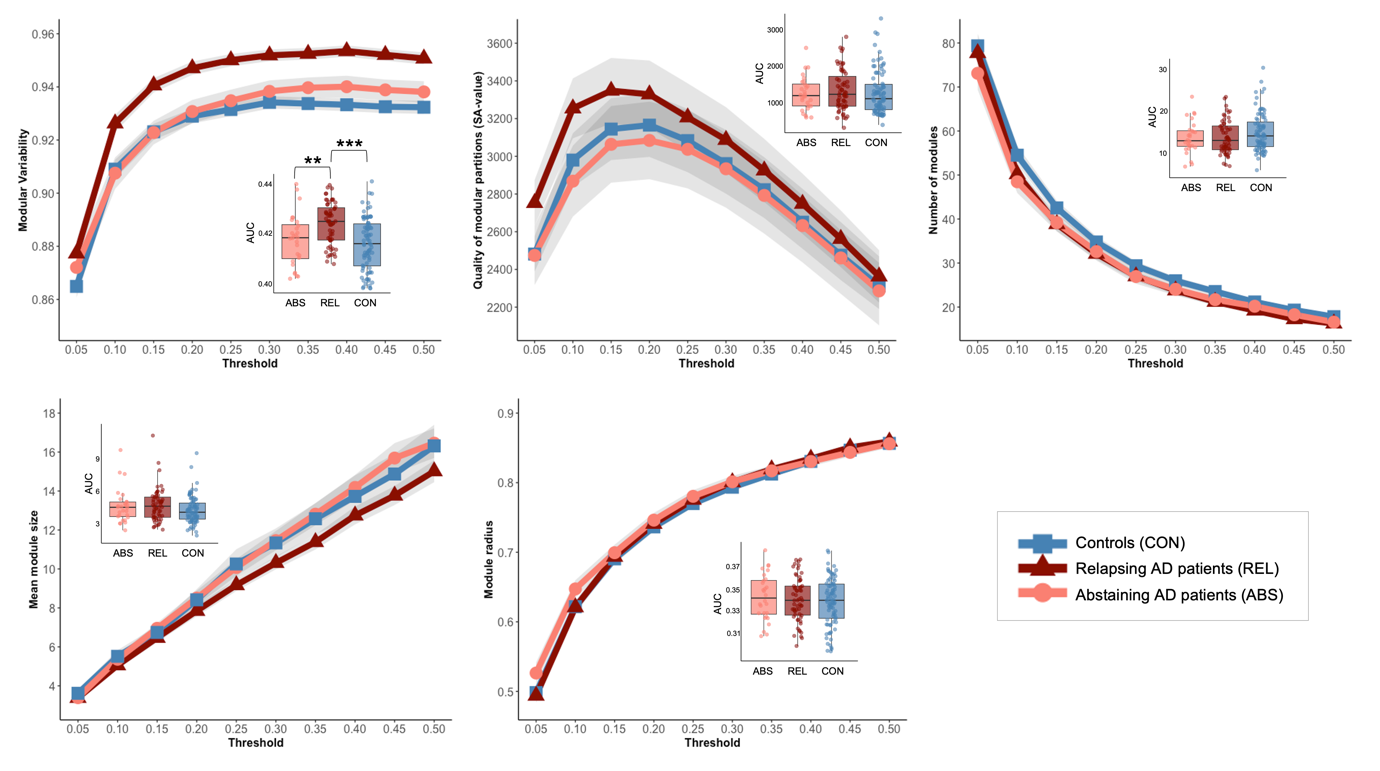


**Figure S4**. Robustness analysis using proportional thresholding. Mesoscale network metrics were recomputed across proportional thresholds (5–50% in 5% increments) to assess the robustness of findings obtained with percolation-based thresholding. For each threshold, we derived the corresponding modular partitions and computed the associated mesoscale network metrics. To obtain a threshold-independent estimate for each metric, we calculated the area under the curve (AUC) across the full threshold range and compared AUC values between groups using the same permutation testing procedure as in the main analysis (10,000 permutations), while controlling for age, sex, site, education, and mean FD. Consistent with the main results, relapsers (REL) showed higher modular variability (MV) compared to abstainers (ABS; *p* = .003) and controls (CON; *p* > .001), whereas no significant group differences were observed for modular partition quality (*p* = .931), number of modules (*p* = .070), mean module size (*p* = .055), or module radius (*p* = .312). Large panels display mean per group across proportional thresholds; inset boxplots show the AUC distributions for each group. ** *p* < 0.1, *** *p* < .001

**References**

1 Danon L, Duch J, Diaz-Guilera A, Arenas A, Comparing community structure identification. J Stat Mech Theory. 2005;E9:P09008. <https://doi.org/10.48550/arXiv.cond-mat/0505245>

2 Amelio A, Pizzuti C. Is normalized mutual information a fair measure for comparing community detection methods? [ASONAM '15: Proceedings of the 2015 IEEE/ACM International Conference on Advances in Social Networks Analysis and Mining. 2015](https://dl.acm.org/doi/proceedings/10.1145/2808797);1584-85. <https://doi.org/10.1145/2808797.2809344>

3 Alexander-Bloch AF, Lambiotte R, Roberts B, Giedd J, Gogtay N, Bullmore E. The discovery of population differences in network community structure: New methods and applications to brain functional networks in schizophrenia. Neuroimage. 2012;59(4):3889-3900. <https://doi.org/10.1016/j.neuroimage.2011.11.035>

4 Liao X, Cao M, Xia M, He Y (2017) Individual differences and time-varying features of modular brain architecture. Neuroimage 152:94-107. <https://doi.org/10.1016/j.neuroimage.2017.02.066>

5 Pedersen M, Omidvarnia A, Shine JM, Jackson GD, Zalesky A. Reducing the influence of intramodular connectivity in participation coefficient. Netw Neurosci. 2020;4(2):416-31. <https://doi.org/10.1162/netn_a_00127>

6 Achard S, Delon-Martin C, Vértes PE, Renard F, Schenck M, Schneider F et al. Hubs of brain functional networks are radically reorganized in comatose patients. Proc Natl Acad Sci USA.. 2012;109(50):20608-13. <https://doi.org/10.1073/pnas.1208933109>

7 Bai L, Yin B, Lei S, Li T, Wang S, Pan Y et al. Reorganized hubs of brain functional networks after acute mild traumatic brain injury. J Neurotrauma. 2023;40(1-2):63-73. <https://doi.org/10.1089/neu.2021.0450>

8 De Pauw R, Aerts H, Siugzdaite R, Meeus M, Coppieters I, [Caeyenberghs](https://pubmed.ncbi.nlm.nih.gov/?sort=date&term=Caeyenberghs+K&cauthor_id=31764388) K et al. Hub disruption in patients with chronic neck pain: a graph analytical approach. Pain. 2020;161(4):729-41. <https://doi.org/10.1097/j.pain.0000000000001762>

9 Böhmer J, Reinhardt P, Garbusow M, Marxen M, Smolka MN, Zimmermann US et al. Aberrant functional brain network organization is associated with relapse during 1-year follow-up in alcohol-dependent patients. Addict Biol. 2023;28(11):e13339. <https://doi.org/10.1111/adb.13339>

10 Smith SM, Fox PT, Miller KL, Glahn DC, Fox PM, Mackay CE et al. Correspondence of the brain's functional architecture during activation and rest. Proc Natl Acad Sci USA. 2009;106(31):13040-5. <https://doi.org/10.1073/pnas.0905267106>

11 Yeo BTT, Krienen FM, Sepulcre J, Sabuncu MR, Lashkari D, Hollinshead M et al. The organization of the human cerebral cortex estimated by intrinsic functional connectivity. J Neurophysiol. 2011;106(3):1125-65. <https://doi.org/10.1152/jn.00338.2011>

12 Schaefer A, Kong R, Gordon EM, Laumann TO, Zuo XN, Holmes AJ et al. Local-global parcellation of the human cerebral cortex from intrinsic functional connectivity MRI. Cereb Cortex. 2018;28(9):3095-114. <https://doi.org/10.1093/cercor/bhx179>

13 Gordon EM, Laumann TO, Adeyemo B, Huckins JF, Kelley WM, Petersen SE. Generation and evaluation of a cortical area parcellation from resting-state correlations. Cereb Cortex. 2016;26(1):288-303. <https://doi.org/10.1093/cercor/bhu239>

14 Power JD, Cohen AL, Nelson SM, Wig GS, Barnes KA, Church JA et al. Functional network organization of the human brain. Neuron. 2011;72(4):665-78. <https://doi.org/10.1016/j.neuron.2011.09.006>

15 Laird AR, Fox PM, Eickhoff SB, Turner JA, Ray KL, McKay DR et al. Behavioral interpretations of intrinsic connectivity networks. J Cogn Neurosci. 2011;23(12):4022-37. <https://doi.org/10.1162/jocn_a_00077>

16 Fan L, Li H, Zhuo J, Zhang Y, Wang J, Chen L et al. The human Brainnetome atlas: a new brain atlas based on connectional architecture. Cereb Cortex. 2016;26(8):3508-26. <https://doi.org/10.1093/cercor/bhw157>

17 Shen X, Tokoglu F, Papademetris X, Constable RT. Groupwise whole-brain parcellation from resting-state fMRI data for network node identification. Neuroimage. 2013;82:403-15. <https://doi.org/10.1016/j.neuroimage.2013.05.081>
